# Spatiotemporal Coordination of Intelligent Autonomous Vehicles in Automated Container Terminals Using Deep Reinforcement Learning

**DOI:** 10.3390/s26216910

**Published:** 2026-10-31

**Authors:** Jia Li, Xinglu Xu, Zijian Guo, Wenyuan Wang, Yun Peng, Zicheng Xia, Yilin Wang

**Affiliations:** 1 State Key Laboratory of Coastal and Offshore Engineering, Dalian University of Technology, Dalian 116024, China; lijiaaa@mail.dlut.edu.cn (J.L.); zjguo@dlut.edu.cn (Z.G.); wangwenyuan@dlut.edu.cn (W.W.); yun_peng@dlut.edu.cn (Y.P.); 20201081274@mail.dlut.edu.cn (Y.W.); 2 Navigation College, Dalian Maritime University, Dalian 116026, China; xiazicheng183@dlmu.edu.cn

**Keywords:** intelligent autonomous vehicle, path planning, platoon strategy, reinforcement learning, automated container terminal

## Abstract

Capacity-limited lanes and intersections constrain the efficiency of intelligent autonomous vehicles (IAVs) in automated container terminals (ACTs). Although onboard sensing and traffic monitoring can provide vehicle state information, translating this information into coordinated routing and intersection decisions remains challenging. Therefore, this study proposes a two-layer coordination framework that integrates path planning with adaptive platoon size control at intersections. The first layer develops a path planning method based on a conflict point network. By dynamically updating continuous timelines of conflict points to record spatiotemporal occupancy states, this method establishes key feasibility constraints that ensure collision-free routing and minimize the task completion time of IAVs. The second layer employs a Deep Q-Network (DQN) to adjust intersection platoon sizes according to current platoon configurations and traffic performance feedback. The framework operates on processed traffic states that could be supplied by vehicle localization and monitoring systems. This learning-driven control mechanism enhances intersection throughput, reduces congestion, and enables adaptive coordination in dynamic operating environments. The proposed framework is evaluated under varying traffic demand scenarios. Compared to traditional rule-based methods, experimental results demonstrate that it reduces average delay by up to 34.45% at isolated intersections and 27.60% in network-wide settings. These findings highlight the effectiveness of the proposed framework in improving IAV operations and offer a scalable solution to support future port automation and intelligent transportation management.

## 1. Introduction

**Figure 1:**
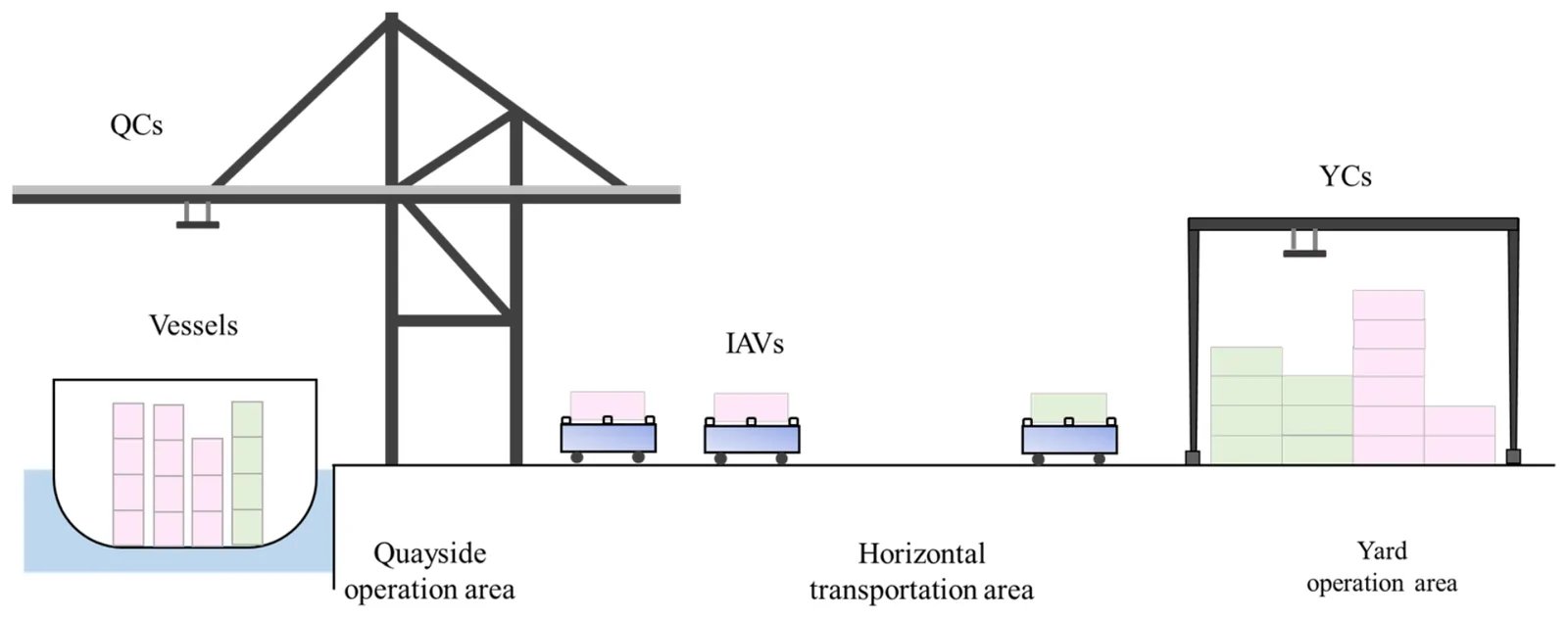
The layout of an ACT.

Shipping is the cornerstone of global trade, accounting for about 80% of global trade [1]. As global trade grows, container terminals are facing increasing operational pressure. To improve efficiency and reduce costs, automation in construction has become a necessary way to enhance competitiveness [2]. Automated container terminals (ACTs) comprise quayside, horizontal transportation and yard operation areas (Figure 1). Horizontal transportation connects the quayside and yard, making the efficiency of horizontal transportation devices (HTDs) critical to overall terminal performance. Although automation has improved the productivity of quay cranes (QCs) and yard cranes [3,4], horizontal transportation remains constrained by congestion and insufficient coordination, particularly under high traffic demand.

The operation of HTDs in ACTs is constrained by limited-capacity resources such as lanes, intersections, and transfer points [5]. When large vessels arrive, a large number of containers must be unloaded and transferred to designated yard blocks within a limited time window. To facilitate management, containers are often grouped by origin and destination, which results in the accumulation of transport demand in specific areas. Consequently, multiple HTDs may be simultaneously allocated to the same loading or unloading point, leading to congestion and queuing at critical nodes. With the trend of larger vessels, these transportation conflicts have become increasingly evident. Intelligent autonomous vehicles (IAVs) are equipped with sensors and onboard computing systems that support perception, communication and autonomous driving. Processed vehicle localization and traffic monitoring information can support the coordination of IAV movements [6]. Compared with automated guided vehicles (AGVs), IAVs offer greater route flexibility and support more complex traffic control strategies [7,8]. These advantages make IAVs a promising solution for improving horizontal transportation efficiency in ACTs.

Nevertheless, this flexibility also introduces new coordination challenges. IAVs must frequently make routing decisions in a dynamic environment where multiple IAVs compete for limited capacity lanes and intersections. The traditional centralized or sequential scheduling methods often fail to balance real-time decision making and global conflict-free coordination. Therefore, it is necessary to study a path planning method with decentralized route computation that considers the operational characteristics of IAVs to achieve safe and coordinated vehicle operation under high traffic demand conditions. Meanwhile, the real-time controllability of IAVs provides new opportunities for optimizing intersection management, especially in terminals with parallel layouts where multiple IAVs frequently traverse intersections to complete transport tasks. When intersections become congested, delays accumulate and affect overall terminal performance. Vehicle observations must therefore support mutually feasible routing and intersection decisions: measured occupancy describes current conditions, whereas future access to conflict points must be coordinated through path reservations and platoon decisions.

Existing studies mainly address path planning and intersection control as separate problems [9,10,11]. Path planning methods typically ensure collision-free routing at the global level, while intersection control studies focus on local coordination and throughput optimization. However, in high-density dynamic terminal environments, these two aspects are intrinsically coupled: global routing decisions directly influence intersection states, while intersection control feedback conversely impacts vehicle routing feasibility. Therefore, this study develops an integrated framework adaptive platoon size control at intersections into the overall path planning process. By establishing a space-time network to represent potential vehicle conflicts and formulate key operational constraints (conflict point occupation, link capacity, and IAV safety buffers), this framework achieves collision-free path planning while significantly enhancing intersection operational efficiency in high traffic scenarios. The contribution of this paper mainly lies in the following aspects:

(1) An integrated coordination framework for IAVs in ACTs is proposed, in which a space-time path planning layer and an adaptive platoon size control layer at intersections are coupled through shared occupancy timelines.
(2) A path planning algorithm with decentralized route computation and centralized reservation management is developed, incorporating timeline-based conflict avoidance. Additionally, a Deep Q-Network (DQN)-based platoon size control strategy is introduced to adaptively determine platoon sizes according to real-time traffic states, thereby enhancing intersection throughput efficiency.
(3) A simulation environment reflecting actual port operations is constructed. The results show that the framework ensures conflict-free routing with millisecond-level computation and reduces the average delay of IAVs by up to 34.45% at isolated intersections and 27.60% at the network level under high traffic demand.

The structure of this paper is as follows: Section 2 gives a review of relevant literature. Section 3 formulates the space-time network based on traffic motion conflict and some specific settings. Section 4 introduces the integrated method for path planning and intersection control for IAVs. Section 5 shows the results of numerical experiments. Section 6 summarizes the main conclusions and future research discussions.

## 2. Literature Review

This section summarizes the existing literature. Section 2.1 introduces the path planning problems for different HTDs. Section 2.2 presents the application of the platoon strategy in intersection control. Section 2.3 reviews the sensing technologies that provide vehicle state information for coordination in automated terminals.

### 2.1. Path Planning Problems in ACT

Path planning for HTDs is a critical process in ACTs, involving the design of efficient routes for vehicles from their initial or starting points to specified destinations, aiming to optimize aspects such as minimal travel time or path length. The results of this planning significantly influence the terminal’s transportation and overall operational efficiency [12].

Numerous studies have explored this topic. Hu et al. [13] proposed a collision-prevention method using reservation schedules, which anticipates potential node conflicts and incorporates waiting windows to prevent deadlocks. Wang and Zeng [14] applied a mixed integer programming (MIP) model to address conflict-free path planning for bidirectional paths in ACTs, optimizing both routing and task sequencing. Hu et al. [12] proposed a multi-intelligence reinforcement learning method to solve the AGV path planning problem for ACTs, which considers the characteristics of AGVs and employs an integer planning model (IP) to determine the shortest paths. Beyond path planning, container transport efficiency in ACTs is also influenced by synchronizing multiple equipment resources [15]. Recent studies have explored the integration of HTDs with other equipment. Yang et al. [16] developed a bi-level programming model for joint scheduling of AGVs, QCs, and rail-mounted gantry cranes, aiming to reduce idle time through coordinated path planning. Similarly, Zhong et al. [17] presented a mixed integer planning model based on path optimization and integrated scheduling to implement the integrated scheduling of multi-AGVs with conflict-free path planning.

While most HTD research has focused on AGVs, the advancement of unmanned technology has recently sparked interest in exploring IAVs. Based on the characteristics of IAVs, Gelareh et al. [8] introduced a pairing/unpairing collaboration strategy for dynamically adjusting IAV task allocation and path selection. Niu et al. [18] investigated the issue of coordinated scheduling in the context of automatic double cantilever rail cranes, intelligent guided transport vehicles (IGVs), and external trucks within a yard that operates under a multi-point loading and unloading mode in a U-shaped automated terminal. The researchers also devised a dynamic balancing strategy to address the path-planning problem associated with IGVs. Yang et al. [19] further modeled the collaborative scheduling of IGVs and cranes by minimizing resource occupation times in the ship unloading process.

In summary, most existing HTD path planning studies adopt centralized optimization frameworks to achieve conflict-free routing. Although these approaches can guarantee solution quality, their scalability and real-time adaptability are significantly limited in large-scale and high-density terminal networks, where the number of vehicles and potential conflicts grows exponentially. Furthermore, most models are static and cannot dynamically update routing decisions based on real-time operational changes. To overcome these limitations, this study proposes a space-time path planning method with decentralized route computation that continuously updates node availability and conflict information through shared occupancy timelines. This method enables each IAV to generate its own conflict-free route without a central optimizer jointly planning all routes, enhancing scalability and robustness under dense traffic conditions.

### 2.2. Platoon Strategy in the Intersection

**Figure 2:**
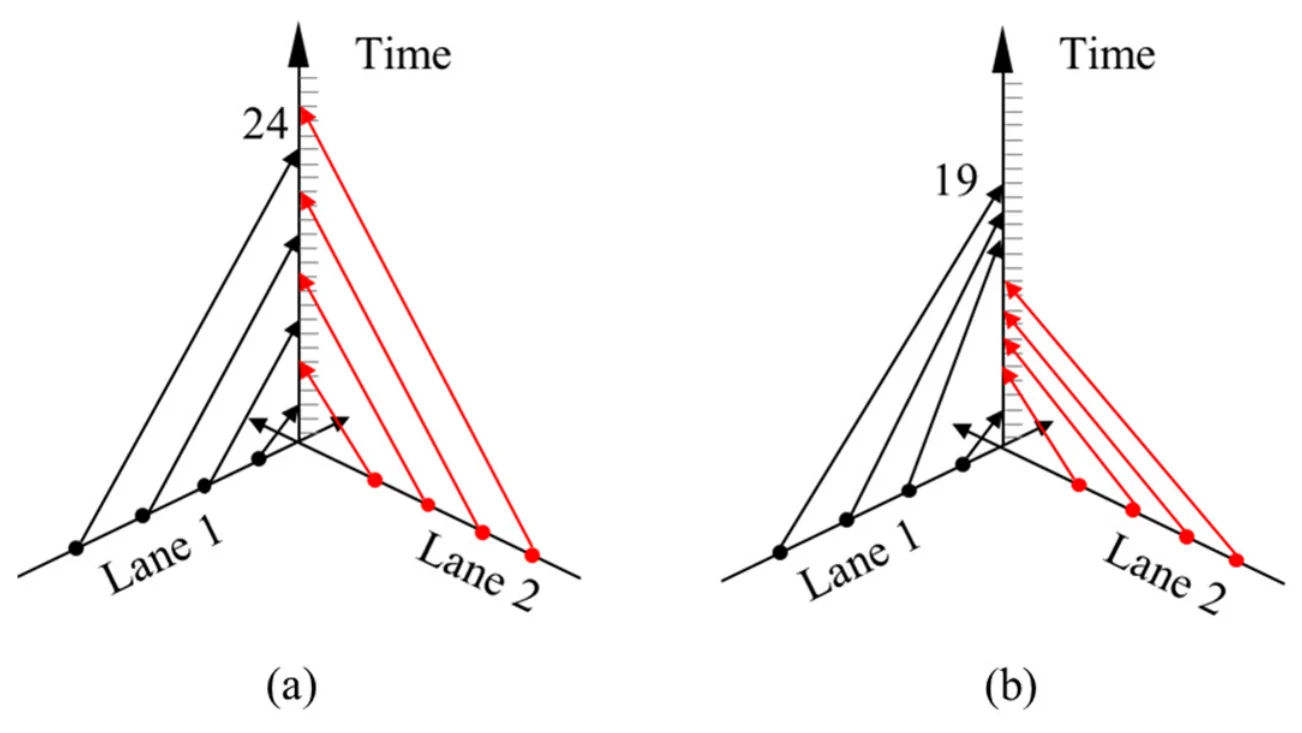
Passing sequence of IAVs from two lanes at a conflict point: (**a**) without platoon control (FCFS); (**b**) with platoon control. The vertical axis denotes the passing time at the conflict point.

Platooning is a concrete implementation strategy that can be used for automated intersection control [20]. It can potentially provide significant advantages for road traffic, including enhanced traffic safety, increased road capacity, and reduced fuel consumption [21]. However, it is essential to recognize that achieving cooperation among all automated vehicles (AVs) within a system may be impractical or unnecessary. Performing route coordination for AVs at intersections can substantially improve overall traffic flow dynamics [22]. Figure 2 illustrates a comparative scenario with and without platoon control at an intersection, further illustrating the benefits of the platoon strategy. In this scenario, we consider four IAVs positioned on two lanes. To ensure simplicity, this paper establishes a minimum time gap of 3 s between the merging of two vehicles and a minimum time interval of 2 s between two following IAVs. Under the First Come First Served (FCFS), vehicles must alternate their passage, resulting in a total travel duration of 24 s for all vehicles. Conversely, the platoon strategy enables continuous passage for vehicles in the same lane, reducing the total time to 19 s.

Platoon strategies have been extensively studied. Bhoopalam et al. [23] provided a comprehensive overview of platoon applications in recent years, delving deeply into the driving benefits of platooning. Wang et al. [24] explored the positive impacts of AV platoons, focusing on a centralized control strategy that enhances safety and fuel efficiency. Mu et al. [25] developed an optimal trajectory plan for the safe and efficient merging of two platoons at intersections, although their analysis did not consider the teaming process. In port environments, research on platooning remains limited. Chen et al. [26] formulated a mixed-integer second-order cone programming model for platoon-based scheduling of autonomous trucks between terminals, jointly optimizing truck dispatching, platoon formation, and travel speed. Xue et al. [27] developed a semi-autonomous platoon scheduling model to reduce fuel consumption and labor costs in container drayage. You et al. [28] developed a comprehensive framework to simulate the truck platoon mode in addressing the local container drayage problem, evaluating the effect of maximum platoon size on overall costs. For IAV dispatching in the yard, Dong et al. [29] gave an initial plan to dispatch vehicles in the IAV-based CT, which enabled the IAV to join or leave a platoon on the way to its destination, then disengage from the platoon and put down the container after arriving at the designated location.

Despite these advances, most existing platoon research in port contexts does not integrate dynamic routing algorithms or address real-time coordination at intersection bottlenecks. To address this gap, this paper introduces a novel intersection control method for ACTs, which leverages platoon strategies to dynamically optimize intersection traffic flow and alleviate congestion. By integrating a decentralized space-time routing algorithm, the proposed approach enables conflict-free routing and adaptive local coordination for IAVs operating under high traffic demand.

### 2.3. Sensing for Vehicle Coordination in Automated Terminals

A third body of work concerns the measurements on which coordination depends. On the vehicle side, centimetre-level positioning of industrial vehicles can be achieved by fusing odometry, inertial and exteroceptive measurements [30], and learning-based schemes can adaptively weight LiDAR- and vision-based estimates [31]. On the infrastructure side, roadside LiDAR, cameras and camera–radar fusion provide lane-level vehicle detection and tracking at intersections [32,33]. These studies establish what an ACT sensing system can realistically deliver in terms of accuracy, range and update rate, but they stop at the perception output and do not address how the resulting observations should drive routing and right-of-way decisions. The framework proposed in this paper is positioned at exactly this interface: it consumes processed vehicle motion and passage information together with planning and control records to support path planning and platoon coordination. In the present study, sensing and communication are assumed to be ideal; their accuracy and latency are not modeled explicitly, and their possible effects are discussed as a limitation.

## 3. Problem Statement

### 3.1. Problem Description

In parallel layout ACTs, IAVs are responsible for horizontal container transportation between QCs and storage yards. The dense grid structure of the internal road network creates frequent intersections that become bottlenecks where multiple vehicles converge, making conflicts and congestion unavoidable. The scheduling of IAVs in this environment is particularly challenging due to three factors:

Multi-path conflicts: when IAVs from different QCs or yard blocks arrive at the same intersection simultaneously, intersection conflicts occur, and one vehicle must wait.

High traffic flow: ship operations generate intensive container flows within short intervals, leading to multiple IAVs occupying the same road segments and queuing and delays.

Dynamic traffic demand: the stochastic nature of container tasks makes intersection flows highly variable, and static control rules fail to adapt to real-time changes, reducing overall operational efficiency.

Based on these characteristics, the research problem in this paper is formally defined as the joint optimization of conflict-free path planning and adaptive platoon size control at intersections for IAVs under dynamic traffic conditions. The objective is to minimize total delays and waiting times by ensuring the spatiotemporal feasibility of global routes while improving local intersection efficiency. In this framework, all potential conflict points in the road network are modeled as spatiotemporal resources with limited capacity, requiring IAVs to satisfy both occupancy and safety buffer constraints when passing through.

Path planning determines vehicle arrival patterns at intersections, while intersection control decisions provide feedback by adjusting feasible time windows, thereby ensuring the integration of network-wide feasibility with local efficiency.

The following assumptions are made in this study:

(1) Level of automation. All IAVs are assumed to have Level 4 (high driving automation) capabilities and to perform driving tasks autonomously under the specified operating conditions within the terminal horizontal transportation area, without human driving intervention. Only normal automated operation is considered; remote takeover, manual driving, vehicle failures and degraded automation are excluded.
(2) Information availability and communication. IAVs are assumed to exchange vehicle states and passage reservation information with a terminal coordination server through vehicle-to-infrastructure (V2I) communication. The server centrally maintains shared conflict-point occupancy and reservation records for path planning and platoon size control. Required information is assumed to be available and consistent at the corresponding decision epochs; transmission latency, packet loss and communication outages are not explicitly modeled.
(3) Traffic composition. The modeled traffic stream consists exclusively of IAVs of the same vehicle model, corresponding to an IAV proportion of 100%. During operations, the studied horizontal transportation area is assumed to be segregated from landside traffic, with external trucks, manually driven vehicles, and service and maintenance vehicles excluded from the modeled road network.

### 3.2. The Space-Time Network Based on Traffic Motion Conflict

In traditional network modeling, nodes typically represent intersections, and arcs symbolize roadway segments [34]. However, this method falls short of capturing the complex motions of IAVs within the complex traffic environment of ACTs. This paper developed a conflict point network, where nodes represent critical conflict points, such as intersections and loading/unloading areas, and arcs represent lane segments. IAVs are modeled as road vehicles traveling on directed lanes within the terminal, rather than on physical tracks or embedded guide paths. Each arc represents a permitted movement along a road-lane segment, with its direction given by the traffic organization of the terminal.

**Figure 3:**
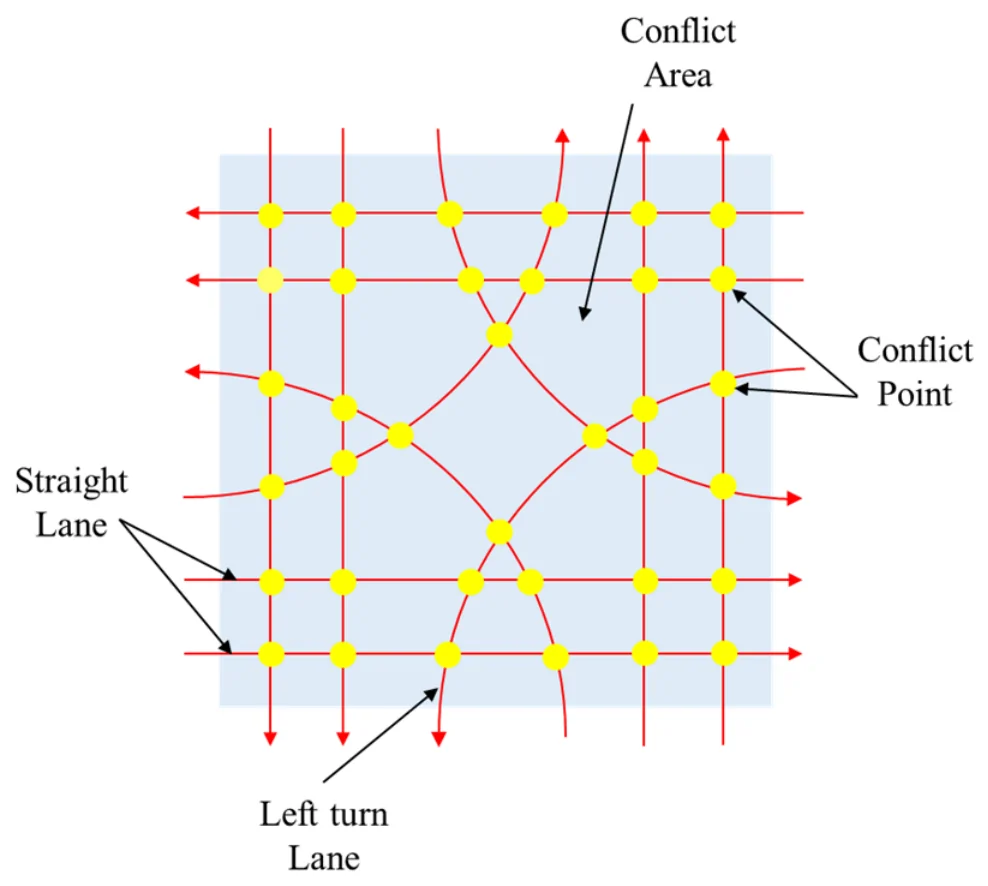
Illustration of an intersection in the network.

Building on this structure, we construct a space-time graph 𝒢 = (*N*,*A*), where *N* is the set of conflict points, and *A* ⊆ *N* × *N* represents feasible lane segments between conflict points. In the graph, the node *n*_1_ is called the upstream node to node *n*_2_, if there is an arc (*n*_1_, *n*_2_) ∈*A*. The upstream node (arc) is denoted as *n*_1_ → *n*_2_. Accordingly, node *n*_2_ is called the downstream node to node *n*_1_. Figure 3 shows the network representation of an intersection in the yard. Unlike general path planning networks with arbitrary origins and destinations, the path planning of IAVs in ACTs must align with operational workflows. Therefore, we designate fixed loading/unloading locations as the origin and destination nodes. Furthermore, each conflict point is associated with a time-dependent occupancy window, reflecting operation-specific dwell times. For example, loading/unloading points typically require longer service times than other nodes, i.e., *τ*^1^ < *τ*^2^.

### 3.3. IAV Conflict Type and Safety Buffer

**Figure 4:**
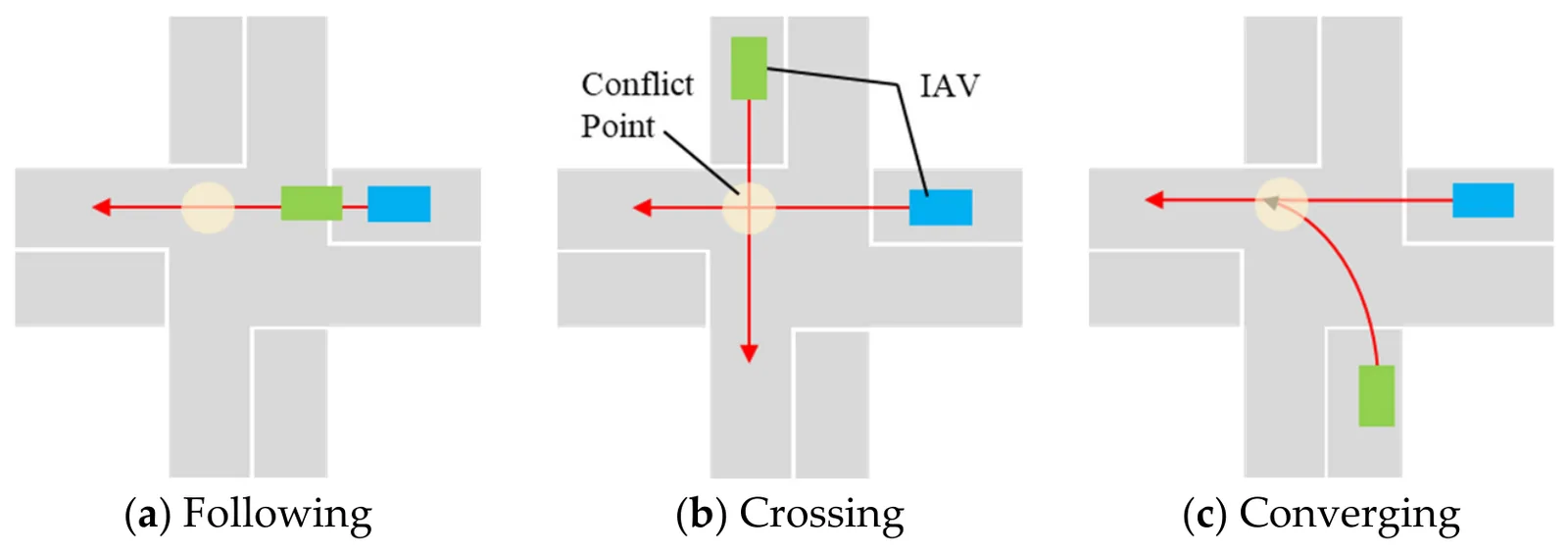
Three types of IAV conflicts at points.

The complexity of traffic control stems from the conflicting dynamics between different traffic movements. For instance, as illustrated in Figure 4, when two sequential IAVs pass through a conflict point within an intersection, there are typically three types of conflict: crossing, converging, and following state [35]. To ensure safety among IAVs, the safety time distance that needs to be maintained between IAVs varies for different conflict types, which is also called the safety buffer. Notably, a larger safety buffer is necessary when IAVs are crossing, followed by the converging state, while the following state demands the smallest safety buffer. In this paper, following conflicts may occur at all conflict nodes except the starting and ending nodes, denoted by *N^c^*, whereas crossing and converging conflicts occur only at specific nodes within intersections, denoted by *N^m^*. Consequently, we introduce two levels of safety buffers: $b_{i}^{1}$ and $b_{i}^{2}$. Here, $b_{i}^{1}$ denotes the safety buffer in the following point, while $b_{i}^{2}$ denotes the safety buffer at crossing and converging points.

### 3.4. Conflict Avoidance Method

To avoid potential collisions, this study introduces a dynamic conflict avoidance mechanism based on continuous space–time timelines. Each node in the conflict point network can only be occupied by one IAV at a time, and a continuous timeline *T_n_* is established for recording the occupancy of each node *n* ∈ *N*, represented as *T_N_* = {*T*_1_,*T*_2_,…, *T_n_*}. In addition, if an IAV *k* arrives at a node, the node occupation time will be generated. Different node types correspond to different occupancy times.

**Figure 5:**
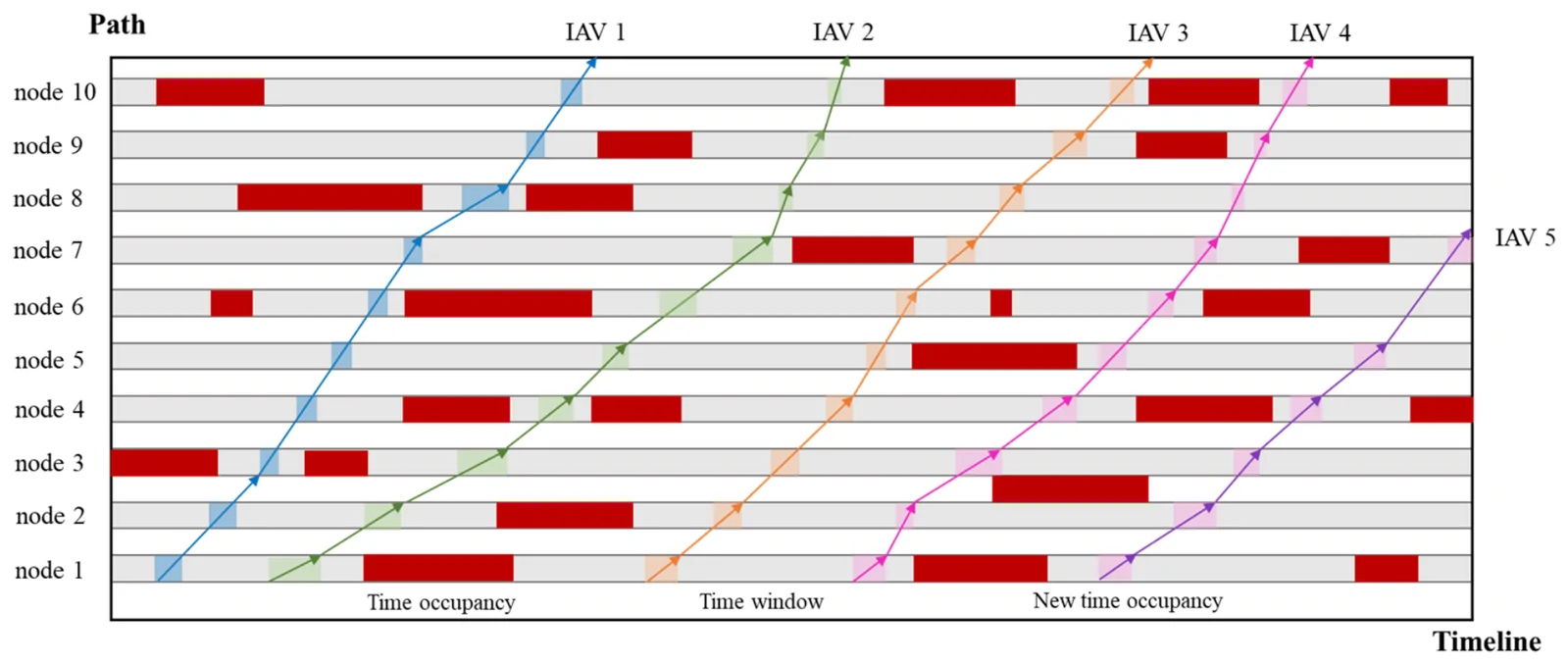
Conflict avoidance considering the time window.

As the IAV enters the network, the occupancy timeline of the accessed node is dynamically updated. At any given moment, the current occupancy status of all nodes can be obtained, enabling path planning algorithms to determine feasible arrival and departure times in real time. The earliest departure time (EDT) for vehicles at each node is dynamically adjusted based on the IAV’s current motion state and the temporal availability of subsequent nodes, thereby maintaining collision-free operation. The specific process of conflict avoidance is illustrated in Figure 5. Specifically, when an IAV departs from the current node to the next node, it can continue to travel at the original speed or a higher speed if no other IAVs occupy the corresponding segment on the timeline of the next node. Otherwise, the IAV must decelerate or postpone its EDT to prevent overlapping occupancy intervals.

## 4. Methodology

### 4.1. Method Framework

This paper presents an integrated method combining conflict-free path planning with adaptive platoon size control at intersections through a two-layer framework. This framework consists of a path planning layer and a platoon size control layer, which are integrated through a timeline updating mechanism. From an implementation perspective, the framework operates downstream of vehicle localization and traffic monitoring systems. Processed observations could provide current vehicle motion and passage information, whereas task requirements and future node reservations remain outputs of operational management and planning, respectively. The conflict point timelines should therefore be distinguished from raw sensor measurements: they record scheduled occupancy intervals and are updated through the routing and platoon size control processes. The present study evaluates these coordination processes using simulated traffic states without developing a new sensing or localization algorithm.

**Figure 6:**
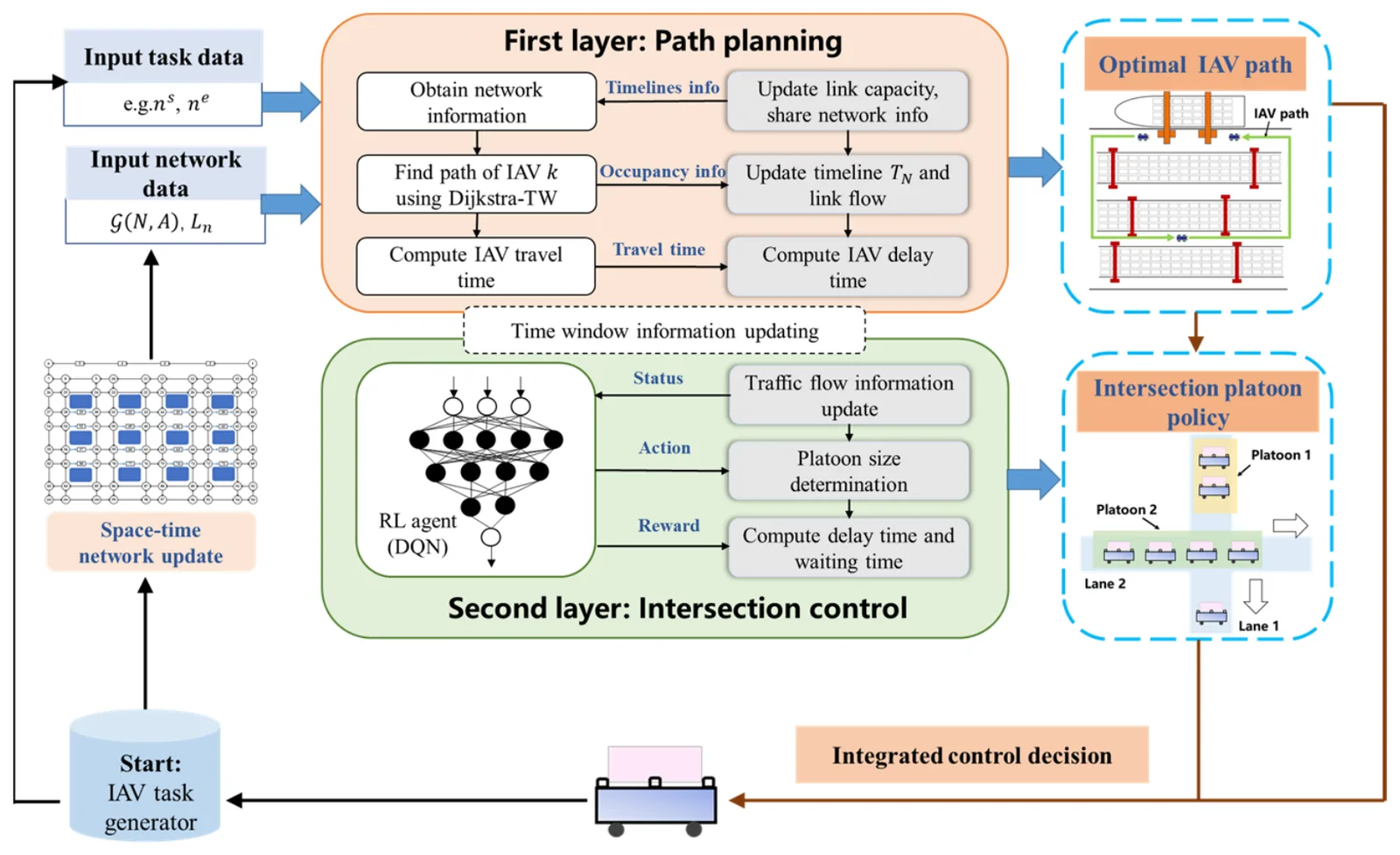
Framework of the integrated method.

Figure 6 illustrates the implementation process of the proposed method. In the first layer, IAV routes are determined on a space-time network where conflict points are modeled as capacity-constrained resources with time-window constraints. The Dijkstra-TW algorithm constructs routes sequentially for individual IAVs using the network topology and the shared occupancy timelines *T_N_*, which are maintained by the terminal coordination server. During each path search, existing reservations are treated as fixed inputs. Once a route is selected, its occupancy reservations are recorded in *T_N_* before the next routing request is processed (see Section 4.2). In the second layer, a DQN-based platoon size control model is established. The model uses current platoon configurations and traffic performance feedback to adjust platoon sizes, with the aim of reducing average waiting and delay times (see Section 4.3).

At the beginning of each control step, a separate adjustment is selected for each controlled lane using the same pre-update network state *S_t_*. The adjustments and the associated updates to unreserved conflict-point time windows are synchronized across the controlled intersections before subsequent routing requests are processed. Each newly released task is routed once using Dijkstra-TW and the current *T_N_*. Confirmed reservations, including future passage times, take precedence over new platoon settings and remain unchanged. Consequently, subsequent platoon size adjustments do not revise previously confirmed routes or passage times. With these interactions, the two-layer framework coordinates network-wide routing with local platoon size control under the stated operational assumptions.

### 4.2. First Layer: Algorithm Design of Path Planning

#### 4.2.1. Settings and Hypotheses

Based on the detailed description of the integration problem in the previous section, the other assumptions are as follows:

(1) The initial position and task assignment of each IAV are predetermined and known.
(2) To develop a corresponding operation plan, QCs and RMG cranes can perform the tasks as planned, and to ensure that the QCs are fully utilized, this paper assumes that no QCs are waiting for the IAVs and no IAVs are waiting for the QCs.

The notation used in the model is summarized in Table A1 (Appendix A).

#### 4.2.2. Model Formulation

This section presents a mathematical formulation that formalizes the spatiotemporal feasibility constraints governing IAV operations. Based on the parameter settings defined above, the path planning constraints for each IAV *k* are formulated, with the objective function of minimizing the time for IAV *k* to complete a transport task. The objective function is:

(1) $$\min\left(a_{k,e_{k}}-T_{k}^{\mathrm{gen}}\right)$$

IAVs are planned sequentially on a first-come-first-served (FCFS) basis according to their task release times $T_{k}^{\mathrm{gen}}$. When IAV *k* is planned, Equation (1) is minimized subject to Constraints (2)–(10), in which the arrival and entry times of the previously planned IAVs $k^{\prime }\in K_{k}^{p}$ are fixed to the time windows recorded in *T_N_*. Hence, the path planning layer pursues the earliest arrival of each IAV, whereas network-level traffic efficiency is improved by the intersection control layer, which adjusts the consecutive passing opportunities of different directions at intersections (Section 4.3).

Constraint (2) ensures that the arrival time of IAV *k* at its start node is not earlier than the release time of the corresponding transport task.

(2) $$a_{k,s_{k}}\geq T_{k}^{\mathrm{gen}},\forall k\in K$$

Constraints (3) and (4) ensure that the travel time of the IAV is consistent with its load condition. Specifically, the arrival time at the next node must not be earlier than the arrival time at the current node plus the service time and the minimum travel time. This minimum travel time is determined by the arc length and the corresponding maximum speed (*v*_empty_ or *v*_load_), and 1_{⋅}_ is the indicator function, which equals 1 if the condition in braces holds and 0 otherwise.

(3) $$a_{k,j}\geq a_{k,i}+\tau_{i}+\frac{d_{\mathrm{ij}}}{v_{\mathrm{empty}}}-M\left(1-\delta_{k,\mathrm{ij}}\cdot 1_{\left\{s_{k,\mathrm{ij}}=\mathrm{empty}\right\}}\right),\;\forall k\in K,(i,j)\in A$$

(4) $$a_{k,j}\geq a_{k,i}+\tau_{i}+\frac{d_{\mathrm{ij}}}{v_{\mathrm{load}}}-M\left(1-\delta_{k,\mathrm{ij}}\cdot 1_{\left\{s_{k,\mathrm{ij}}=\mathrm{load}\right\}}\right),\;\forall k\in K,(i,j)\in A$$

Roads in the port differ in width and number of lanes, and therefore in the number of vehicles they can accommodate. Handling lanes are generally one-way corridors where IAVs perform loading and unloading operations, and these narrow lanes are prone to congestion and potential collisions. Constraint (5) ensures that only one IAV can occupy each handling lane arc at a time. When one vehicle travels on the handling lane arc, another must wait until the preceding vehicle has completely left before it can proceed. This prevents congestion and collisions in the narrow operating area.

(5) $$\begin{array}{c} \beta_{k,\mathrm{ij}}\geq a_{k^{\prime },j}-M\left(1-w_{k,k^{\prime },(i,j)}\right)-M\left(2-\delta_{k,\mathrm{ij}}-\delta_{k^{\prime },\mathrm{ij}}\right)\\ \beta_{k^{\prime },\mathrm{ij}}\geq a_{k,j}-Mw_{k,k^{\prime },(i,j)}-M\left(2-\delta_{k,\mathrm{ij}}-\delta_{k^{\prime },\mathrm{ij}}\right)\\ \forall \left(i,j\right)\in A^{\mathrm{hand}},k\in K,k^{\prime }\in K_{k}^{p} \end{array}$$

Constraint (6) ensures that the departure time of the node is consistent with the entry time of the adjacent arc. When the IAV finishes its operation at the node, its entry time to the next arc must match the completion time at that node.

*β_k_*_,*ij*_ ≥ *a_k_*_,*i*_ + *τ_i_* − *M*(1 − *δ_k_*_,*ij*_) *β_k_*_,*ij*_ ≤ *a_k_*_,*i*_ + *τ_i_* + *M*(1 − *δ_k_*_,*ij*_) ∀*k* ∈ *K*, (*i*,*j*) ∈ *A* (6)

Constraints (7)–(8) introduce safety buffer conditions to avoid collisions and maintain safe operations. Specifically, constraint (7) represents the minimum safety buffer between two IAVs passing through the same conflict node, limited by the following buffer $b_{i}^{1}$. Constraint (8) represents the minimum safety buffer between two IAVs at intersection conflict nodes, limited by the crossing and converging buffer $b_{i}^{2}$.

(7) $$\begin{array}{c} a_{k,i}-a_{k^{\prime },i}\geq b_{i}^{1}-M\left(1-z_{k,k^{\prime },i}\right)\\ a_{k^{\prime },i}-a_{k,i}\geq b_{i}^{1}-Mz_{k,k^{\prime },i}\\ \forall k\in K,k^{\prime }\in K_{k}^{p},i\in N^{c}\cap N_{k^{\prime }} \end{array}$$

(8) $$\begin{array}{c} a_{k,i}-a_{k^{\prime },i}\geq b_{i}^{2}-M\left(1-z_{k,k^{\prime },i}\right)\\ a_{k^{\prime },i}-a_{k,i}\geq b_{i}^{2}-Mz_{k,k^{\prime },i}\\ \forall k\in K,k^{\prime }\in K_{k}^{p},i\in N^{m}\cap N_{k^{\prime }} \end{array}$$

Constraint (9) guarantees path continuity for each IAV.

(9) ∑j:i,j∈A δk,ij−∑h:h,i∈A δk,hi=1,if i=sk−1,if i=ek0,otherwise∀k∈K,i∈N

Constraint (10) defines the valid ranges of all variables.

(10) $$a_{k,i}\geq 0\;\forall k\in K,i\in N\;\beta_{k,ij}\geq 0,\delta_{k,ij}\in \{0,1\},\;\forall k\in K,(i,j)\in A\;z_{k,k^{\prime },i}\in \left\{0,1\right\},\;\forall k\in K,k^{\prime }\in K_{k}^{p},i\in N^{c}\;w_{k,k^{\prime },\left(i,j\right)}\in \left\{0,1\right\},\;\forall k\in K,k^{\prime }\in K_{k}^{p},\left(i,j\right)\in A^{\mathrm{hand}}$$

In summary, Constraints (2) to (10) establish a spatiotemporal feasibility model for IAV path planning. This model includes key constraints related to conflict point occupation, link capacity and safety buffers. Rather than representing a centralized optimization problem, it provides a feasibility framework for decentralized path planning, where each IAV independently determines its path according to the current availability of time windows. These constraints are integrated into the path planning algorithm through the label updating and timeline adjustment mechanisms, enabling conflict-free routing under dynamic terminal conditions.

#### 4.2.3. Dijkstra Algorithm with Time Windows

The IAV path planning problem under fixed time windows is closely related to the Shortest Path Problem with Time Windows (SPPTW), where the objective is to compute a feasible path that satisfies given temporal constraints [36]. Based on the classical Dijkstra algorithm [37], we develop a conflict-free path planning algorithm on the space-time network that incorporates the constraints defined in Section 4.2.2. The proposed method considers the operational characteristics of IAVs in yards, such as safety buffers and speed variations depending on the laden or empty state of the IAV.

**Figure 7:**
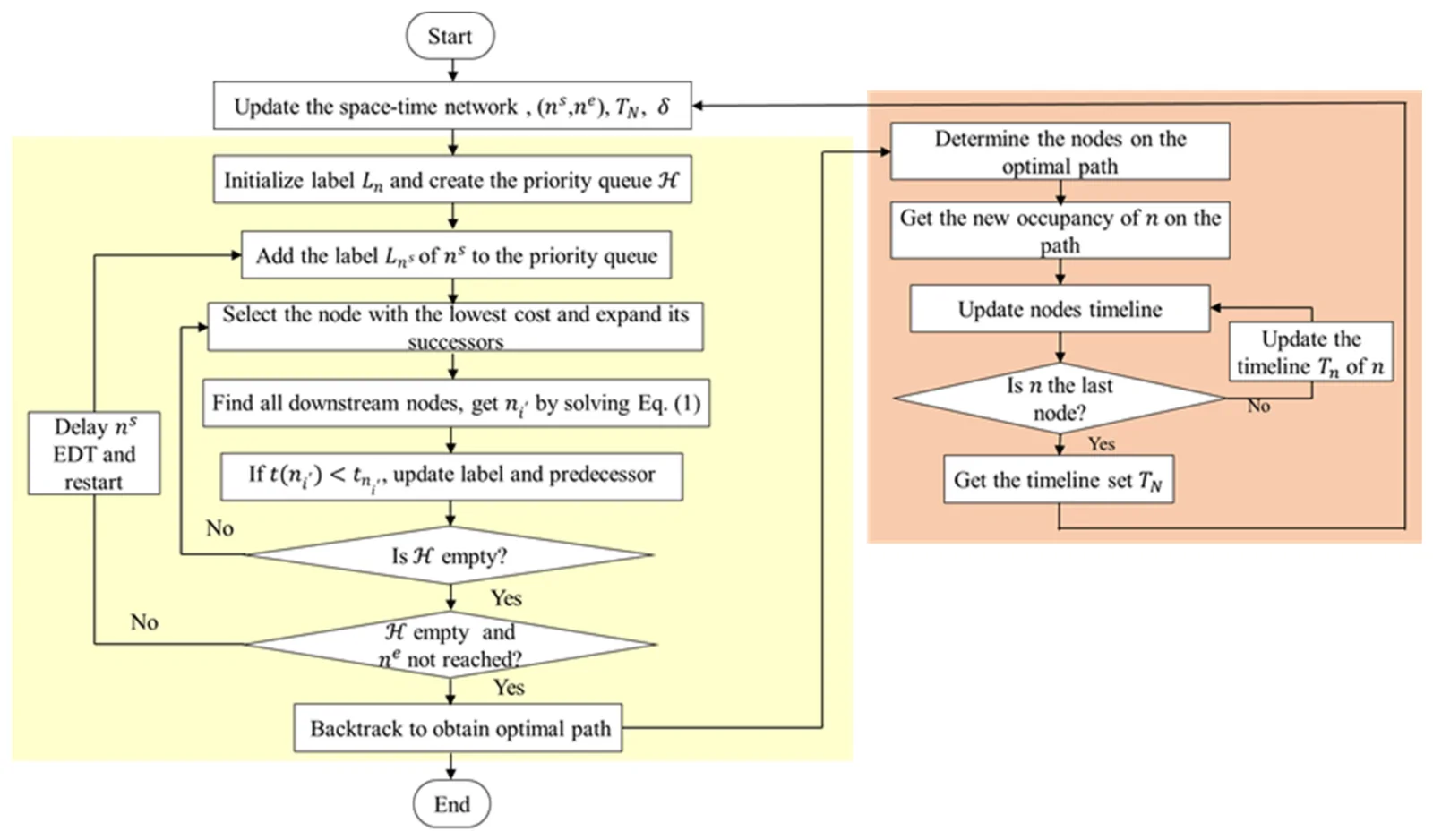
The exact process of Dijkstra-TW.

The proposed Dijkstra-TW algorithm adopts a label-correcting strategy to seek an optimal conflict-free path for the current IAV with respect to the objective in Equation (1), subject to the existing occupancy reservations and spatiotemporal feasibility constraints. Each node *n* in the network is assigned a label $L_{n}=(n,t_{n},T_{n}^{\mathrm{init}},{\mathrm{upstr}}_{n})$. This label encompasses the label cost *t_n_*, the timeline *T_n_ ϵ T_N_*, and the upstream node *upstr_n_*. Here, *T_n_* records the time window usage of node *n*, the ID of the IAV occupying node *n*, and the IAV pass order of *n*. The exact process of Dijkstra-TW is shown in Figure 7. This algorithm accepts four inputs: network 𝒢*^initial^*(*N*,*A*), the starting point and ending point of a task (*n^s^*,*n^e^*), the set of timelines of all nodes in the network *T_N_*, and the step size *δ*. The final output is the optimal path p*.

(1) Update the space-time network

Step 1 Construct initial space-time networks 𝒢*^initial^* ≡ (*N*,*A*) based on the ACT layout and the distribution of IAV conflict points.

Step 2 Update the space-time network based on the current node occupancy information.

(2) Label correcting

Each node is initialized with a label $L_{n}=(n,\infty ,T_{n}^{\mathrm{init}},\mathrm{nil})$, which is added to the priority queue ℋ. The label-correcting process selects the node *n* with the lowest label cost *t_n_* from ℋ and examines its downstream nodes $n_{i^{\prime }}$ using the current node timelines and the spatiotemporal feasibility constraints in Section 4.2.2. If a lower feasible label cost is obtained, the downstream node’s label is updated with the new cost and upstream node information, and the updated label is added back to ℋ. If ℋ becomes empty without reaching the ending point *n^e^*, the earliest departure time (EDT) at the starting point *n^s^* is delayed by the step size *δ*, and the search is restarted. When ℋ is empty and *n^e^* has been reached, the conflict-free path p^*^ is obtained by backtracking from *n^e^* to *n^s^* using the stored upstream node information.

(3) Timeline readjustment

During the path planning process, a route is constructed for each IAV before it enters the network, using the network topology and the current shared reservation records. Therefore, once the IAV path is determined, the occupancy reservations at the corresponding nodes are recorded in *T_N_*. As IAVs are planned sequentially, the occupancy timelines are progressively updated, enabling subsequent IAVs to avoid already reserved time windows during path search.

This update mechanism ensures time-space feasibility and collision-free routing without requiring a centralized joint optimization of all routes; only the shared timelines are maintained centrally.

### 4.3. Second Layer: Platoon Strategy at ACT Intersections

#### 4.3.1. Platoon Size Determination

Vessel berthing schedules and overall port operations significantly influence the efficiency of IAV transportation within port environments. A significant increase in traffic flow concentration typically occurs during the gathering periods preceding ship arrivals, putting considerable traffic pressure on port transport [38]. The path planning algorithm proposed in Section 4.2 is based on decentralized control, which means that each IAV independently makes decisions without accounting for its impact on subsequently planned IAVs. This independent decision-making is problematic during periods of high traffic, as interactions between IAVs can grow significantly, affecting total completion time.

In addition, the terminal layout is characterized by numerous intersections, making traffic management more complex. The extended length and substantial weight of IAVs exacerbate these challenges, leading to congestion at intersections [39]. To address this issue, this study proposes implementing a platoon strategy at each intersection within the yard. This strategy organizes IAVs traveling in the same direction into platoons of various sizes, enhancing their coordinated movement through intersections and adapting to fluctuating traffic volumes. This strategy improves the overall path planning efficiency of IAVs within the ACT network by addressing congestion at crucial intersections.

#### 4.3.2. General Process

In actual ports, the traffic demand is time-varying due to various factors. Therefore, this paper proposes an online optimization framework based on the RL technique to dynamically determine the platoon size according to the real-time traffic demand. Reinforcement Learning (RL) is most suitable for solving decision-making problems. It determines the optimal behavior by interacting with the environment and observing its responses, thereby obtaining the maximum reward [40]. In this paper, RL enables agents to interact with the traffic network in time steps *T* = {1,2,…,*t*}, then select the optimal platoon size at the intersection lane to minimize the waiting time and the delay time of IAVs. This procedure is regarded as a Markov decision process (MDP). The platoon formation process is as follows:

**Step 1** Obtain current traffic flow, time window occupancy information, and IAV paths from the first layer to assess the need for platoon optimization at a specific intersection;

**Step 2** Use the DQN algorithm to determine the optimal platoon size;

**Step 3** Output the platoon size and the adjusted time window allocation and feed this information back to the first layer to ensure that the planned path does not conflict with the platoon strategy.

#### 4.3.3. Agent Design

This section describes states *S_t_*, actions *A_t_*, and rewards *R_t_*.

The state space is represented by three variables that can reflect the traffic state of the platoon: the platoon size on each lane, the average delay and average waiting time of IAVs on the entire network, described as follows:

(11) $$\begin{array}{c} S_{t}=\left\{S_{1}\left(t\right),S_{2}\left(t\right),\ldots \;,S_{i}\left(t\right),\;T_{\mathrm{wait}}\left(t\right),\;T_{\mathrm{delay}}\left(t\right)\right\}\\ S_{i}\left(t\right)=\{L_{1}\left(t\right),\ldots ,L_{8}\left(t\right)\},\;\mathrm{for}\;i\in [1,6] \end{array}$$

where *S_t_* is the total state of the entire network, which is the set consisting of the states of the 6 intersections in the entire ACT network, the average waiting time *T_wait_*, and the average delay *T_delay_*. Herein, the state *S_i_*(*t*) at each intersection is a set of platoon sizes of eight lanes.

The state representation combines controller-maintained platoon sizes with average waiting and delay times that summarize traffic performance. For deployment, the latter quantities could be derived from time-stamped vehicle movement and passage records, together with the corresponding reference travel times. Raw sensor signals are not direct inputs to the DQN.

The action is defined at the lane level as follows:

*a_i_*_,*l*_(*t*) ∈ {−1,0,1}, *i* = 1,…,6, *l* = 1,…,8 (12)

where *a_i_*_,*l*_(*t*) is the action applied to lane *l* of intersection *i*, at time step t; −1 represents the platoon size of this lane minus 1, 0 represents the platoon size is unchanged, and 1 represents the platoon size being increased by 1. At each time step, an action is selected for every controlled lane, and the actions of all lanes are executed simultaneously. The adaptive control is limited to platoon size. The corresponding updates to conflict-point time windows are consequences of platoon size adjustments rather than additional DQN decision variables. Signal control, lane assignment, independent right-of-way allocation among conflicting movements, and explicit intersection capacity allocation are not included in the learned action space.

Rewards are calculated based on the observed changes in average waiting and delay times before and after implementation, as follows:

*R_wait_* = *T_wait_*(*t*) − *T_wait_*(*t* + 1) (13)

*R_delay_* = *T_delay_*(*t*) − *T_delay_*(*t* + 1) (14)

*R_t_* = *R_wait_* + *R_delay_* (15)

where the total reward *R_t_* is the sum of *R_wait_* and *R_delay_*. A penalty is given if the behavior of the agent results in an increase in the value of *R_wait_* or *R_delay_*, and a reward is given if *R_wait_* and *R_delay_* are decreased.

**Figure 8:**
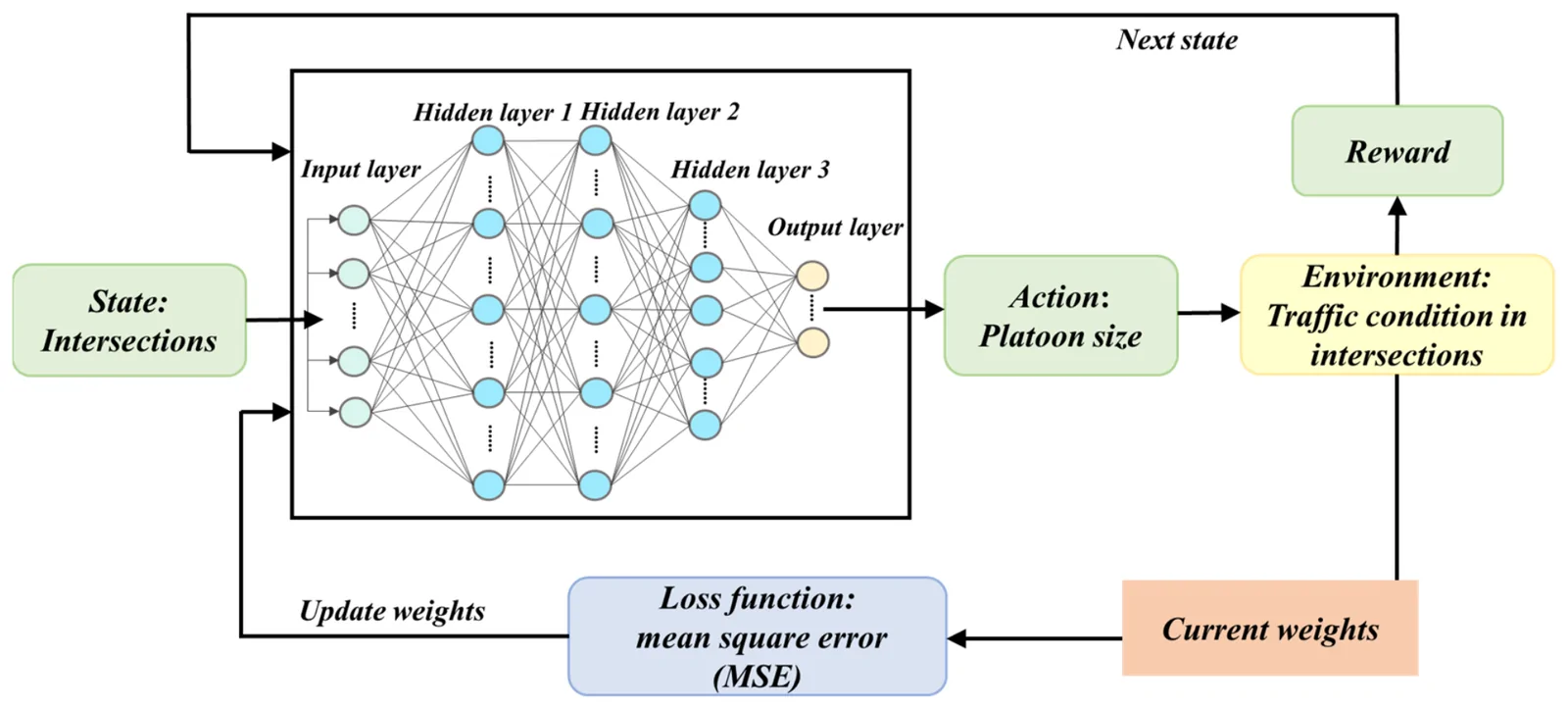
Overview of the DQN algorithm.

Given the complexity and scale of the problem across the entire ACT network, which involves a large number of potential states and actions, this paper adopts a Deep Q-Network (DQN) framework. DQN is a classical deep RL algorithm that employs neural network layers instead of Q-tables used in Q-learning [41]. This approach is better suited for managing the intricate and extensive RL challenges presented by the ACT network. Figure 8 presents an overview of the DQN framework employed.

A strategy *π*: *S* → *A* is a function to return an action *a* ∈ *A* when observing a state *s* ∈ *S*. The action-value function *Q_π_*(s,a) is as follows:

(16) $$\begin{array}{c} Q_{\pi }\left(s,a\right)=E\left\{R_{t}+\gamma R_{t+1}+\gamma^{2}R_{t+2}+\cdots |S_{t}=s,A_{t}=a,\pi \right\}\\ =E\left\{\sum_{k=0}^{\infty }\;\gamma^{k}R_{t+k}|S_{t}=s,A_{t}=a,\pi \right\} \end{array}$$

The optimal π* is used to maximize the expected cumulative reward; it shall satisfy the following equation:

(17) $$Q^{*}(s,a)=\underset{\pi }{\max}\;Q_{\pi }(s,a)$$

where Q* represents the action-value function given the optimal policy π*.

A fully connected deep neural network (DNN) is used to approximate the action-value function. The input layer receives the state vector *S_t_*, and its dimension is determined by the state representation of the corresponding simulation scenario. The network contains four hidden layers with 256, 512, 256 and 128 neurons, respectively. The output layer contains three neurons, each estimating the Q-value of one of the three actions defined in Equation (12). ReLU is applied after each hidden layer, while the output layer uses a linear activation to produce the estimated Q-values.

The study utilizes the Adam algorithm, which iteratively updates the parameters of DNNs to minimize the loss function. The mean square error (MSE) is used as the loss function of the DNN, mathematically expressed as:

(18) $$\mathrm{MSE}(\theta )=\frac{1}{m}\sum_{t=1}^{m}\;{\left\{\left(R_{t}+\gamma \underset{a^{\prime }}{\max}\;Q\left(S_{t+1},a^{\prime };\theta^{\prime }\right)\right)-Q\left(S_{t},A_{t};\theta \right)\right\}}^{2}$$

The experience tuple (*S_t_*, *A_t_*, *R_t_*,*S_t_*_+1_) is stored in the replay memory, and the algorithm proposed in this paper performs updates by uniformly sampling from the memory. Algorithm 1 provides the specific process of DQN implementation.
**Algorithm 1.** DQN-based dynamic platoon size optimization1:Initialize replay memory *D* to capacity2:Initialize action-value function *Q* with random weights *θ*3:Initialize the target network parameters with *θ′* = *θ*4:**For** episode = 1, *M* **do**5:Initialize environment state *S*_1_6:**For** *t* = 1, *T* **do**7:With probability *ε* select a random action *A_t_*8:$\mathrm{otherwise}\;\mathrm{select}\;A_{t}=\underset{a\in A}{\arg\max}Q\left(S_{t},a;\theta \right);$9:Execute action *A_t_* in the simulator and observe reward *R_t_* and the next state *S_t_*_+1_;10:Store transition (*S_t_*, *A_t_*, *R_t_*, *S_t_*_+1_) in *D*11:Sample a random minibatch of transitions (*S_j_*,*A_j_*, *R_j_*, *S_j_*_+1_) from *D*;12:Set yj=Rj, if episode terminates at step j+1Rj+γmaxa′QSj+1,a′;θ′,otherwise;13:Update network parameters *θ* by a gradient descent step on ${\left(y_{j}-Q(S_{j},A_{j};\theta )\right)}^{2}$
14:Every *N*^−^ steps reset *θ*′ ← *θ*15:Set *S_t_* = *S_t_*_+1_16:**End for**17:**End for**

## 5. Numerical Experiments

In this section, numerical experiments are conducted using conflict-free path algorithms and platoon strategies to validate the proposed integrated approach. The experiments were implemented on Windows 10, 12th Gen Intel(R) Core (TM) i7-12700H 2.30 GHz, and 16 GB RAM. The simulations were programmed in Python 3.7.

### 5.1. Experimental Parameters Setting

**Figure 9:**
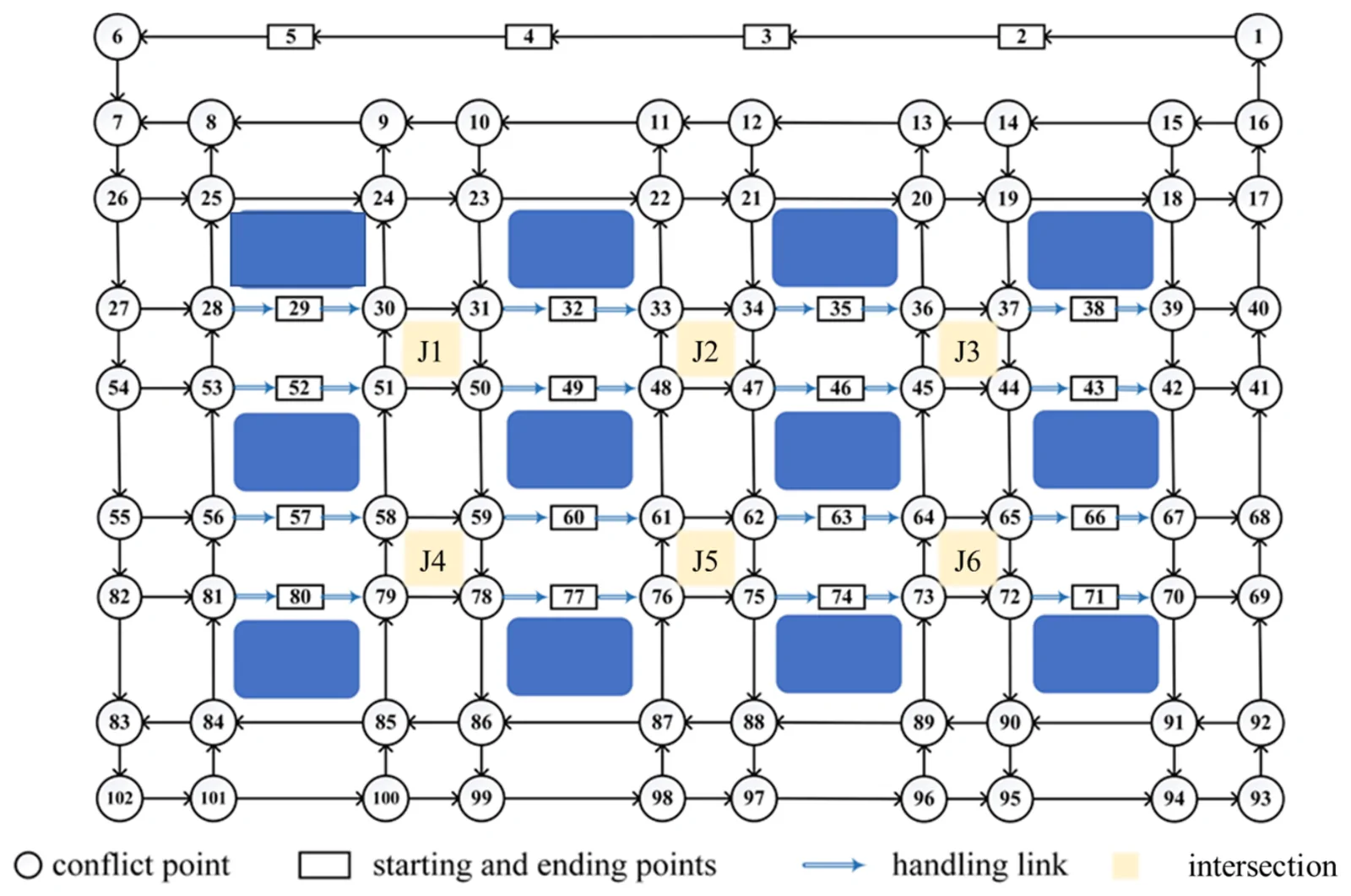
A traffic network based on nodes.

**IAV path network setting.** This experiment uses the operational parameters obtained from an ACT in North China as a reference. Given the specific focus of this paper on paths and intersections within the yard, the layout of the ACT is simplified: The resulting network represents the studied horizontal transportation area rather than a complete reconstruction of the reference terminal. The ACT parameters used in the experiments are summarized in Table A2 (Appendix B). Based on this, a node-based traffic network is constructed (as shown in Figure 9). This network contains 102 nodes, comprising 20 starting (ending) nodes and 82 ordinary conflict nodes. The starting and ending nodes of IAV transport tasks are randomly assigned within the ranges (2–5) and (29, 32, 35, 38, 43, 46, 49, 52, 57, 60, 63, 66, 71, 74, 77, 80), respectively. The second layer is applied to the six intersections J1–J6 marked in Figure 9, i.e., the node groups (30, 31, 50, 51), (33, 34, 47, 48), (36, 37, 44, 45), (58, 59, 78, 79), (61, 62, 75, 76) and (64, 65, 72, 73). Each intersection area comprises multiple conflict nodes. They are the interior intersections of the yard, where the corridors between adjacent block columns cross the operation lanes between block rows. Reflecting real-world operational conditions, the ordinary conflict nodes are designated as *τ*^1^ = 1 s, while the starting (ending) nodes are represented as *τ*^2^ = 15 s. Additionally, safety buffers are implemented as follows: $b_{i}^{1}$ = 1 s, $b_{i}^{2}$ = 3 s/5 s.

**Evaluation scenarios design.** Traffic demand levels are selected with reference to historical operating information collected over a three-month period and engineering judgment to define four simulation demand levels. Demand levels 1 to 3 represent progressively increasing simulation input loads, while Demand 4 is used as a high-demand test scenario. These settings are intended to examine coordination performance under different traffic loads rather than to reproduce a statistically calibrated distribution of observed demand. The demand intensity is denoted by *ρ*. The demand levels vary the transport task input within a fixed network configuration. The results therefore characterize performance under increasing traffic demand in the modeled network. In addition, two simulation scenarios are designed: a full-network simulation to evaluate system-wide performance, and a single-intersection scenario to assess local control effectiveness. For each demand level, three arrival-rate values are randomly sampled from the corresponding interval, each defining one simulation case. Each sampled input rate *ρ* is kept fixed within its case, and the values are listed in Table 1. Within each case, task release times are randomly generated with reference to terminal operating data. In the network scenario, the origin and destination of each task are drawn uniformly at random from the four starting nodes (2–5) and the 16 ending nodes specified in the network description, respectively. The demand ranges in Table 1 are specified separately for these two spatial scales: network demand applies to the modeled network as a whole, whereas single-intersection demand applies to the intersection under evaluation. In the network scenario, arrival patterns and turning movements at individual intersections arise from the generated origin–destination tasks and their planned routes, rather than from an equal allocation of total demand among the six controlled intersections. The total simulation time for each traffic condition is 3600 s. To capture traffic dynamics, the simulation was divided into ten periods of 360 s each.

**Traffic state inputs and sensing interpretation.** The experimental scenarios are defined using terminal layout, task parameters, and the demand settings described above, while dynamic vehicle movements and passage events are generated in simulation. The evaluation focuses on routing and platoon coordination, without assessing sensing accuracy or the effects of sensing and communication impairments.

**Platoon strategy setting**. Although the hyperparameters of RL can affect its performance, they are not the focus of this study. After repeated adjustments, we directly determined the specific configuration of the DQN algorithm, detailed in Table A2 (Appendix B). This configuration prioritizes reliable convergence and accuracy, thus ensuring robust performance under various experimental conditions.

**Evaluation index setting.** Two indicators are employed to assess the effects of the proposed approach. The average delay $\bar{T_{\mathrm{delay}}}$ is used to evaluate overall yard network efficiency, as defined in Equation (19).

(19) $$\bar{T_{\mathrm{delay}}}=\frac{\sum_{k\in K}\left(a_{k}\left(n_{k}^{e}\right)-a_{k}^{f}\left(n_{k}^{e}\right)\right)\;}{K}$$

where *K* is the set of all IAVs, $a_{k}\left(n_{k}^{e}\right)$ is the actual arrival time of IAV *k* on the shortest path, and $a_{k}^{f}$ is the arrival time of IAV *k* traveling at the free-flow speed.

Meanwhile, to evaluate intersection passing efficiency, the average wait time $\bar{T_{\mathrm{wait}}}$ is used as an evaluation metric, delineated by Equation (20).

(20) $$\bar{T_{\mathrm{wait}}}=\frac{\sum_{k\in K}T_{\mathrm{stop}}\;}{K}$$

where *K* is the set of all IAVs and *T_stop_* is the cumulative time for the IAV speed to fall below 0.1 m/s.

### 5.2. Experiments on the Path Planning Algorithm

A comparative experiment was conducted across varying traffic demands to assess the efficacy and real-time performance of the proposed conflict-free path planning algorithm.

**Figure 10:**
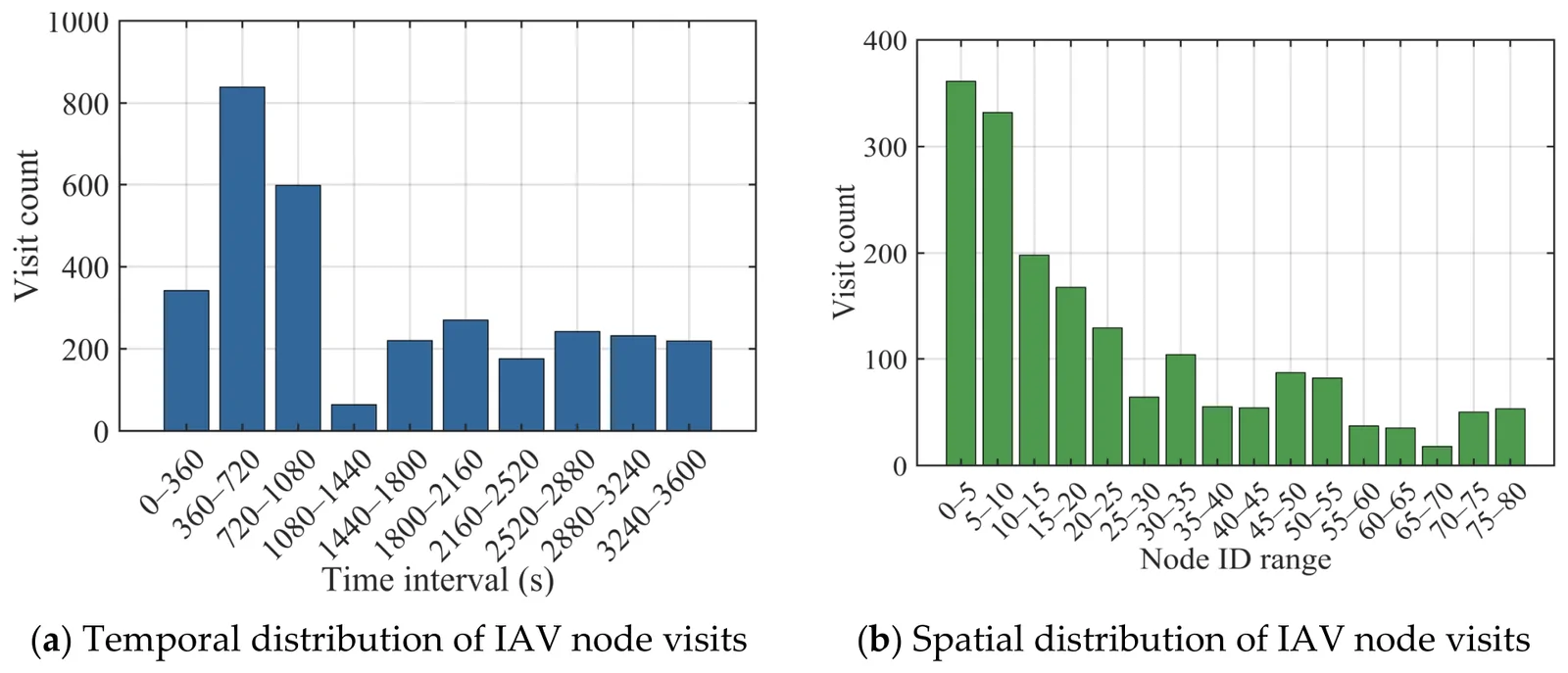
Temporal and spatial distribution of IAV access frequency.

**Figure 11:**
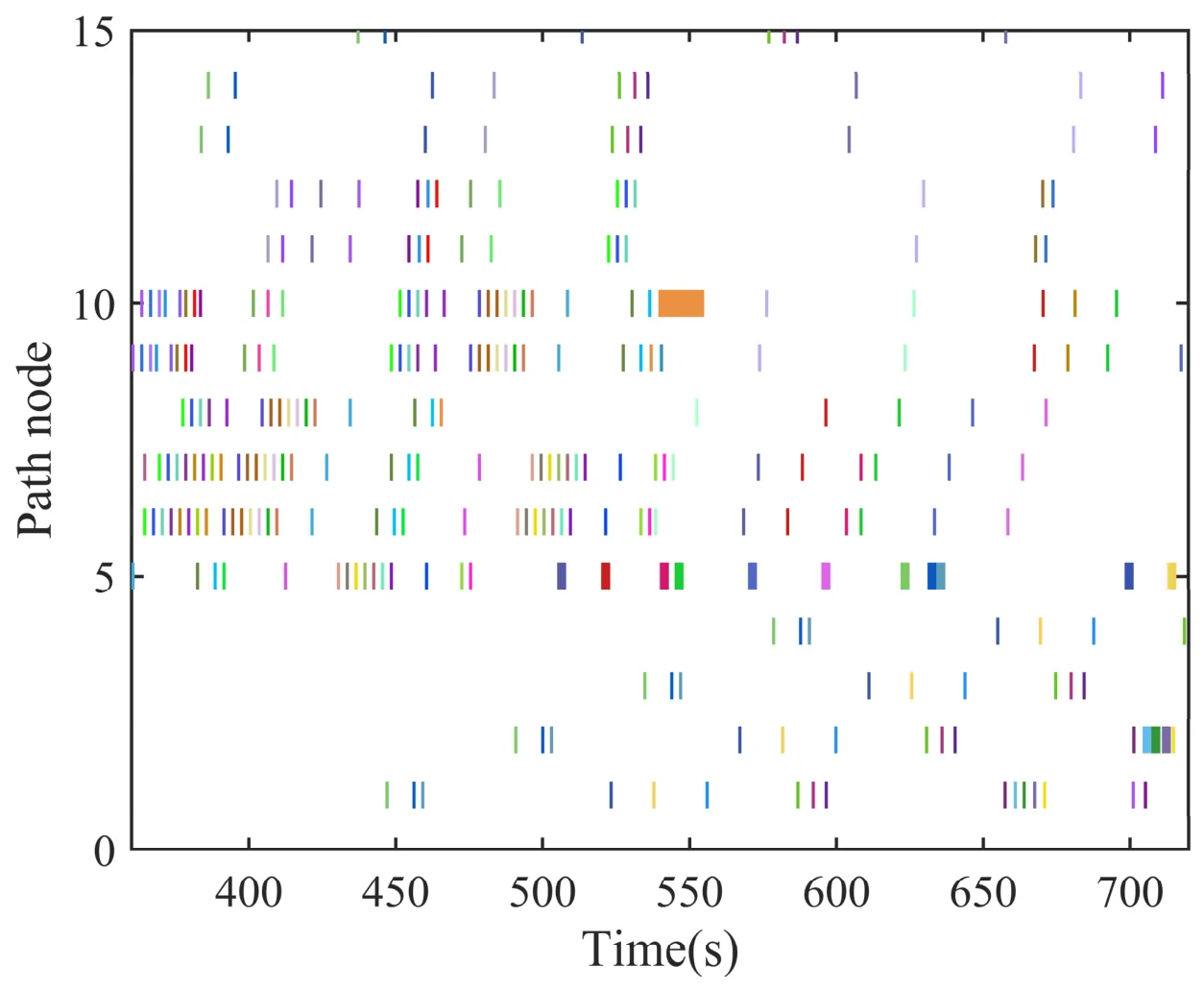
Node occupation timeline of 80 IAVs within [360 s, 720 s] for nodes [1, 15]. Each color represents an individual IAV, and the horizontal extent of each colored segment indicates its occupancy duration at the corresponding node.

**Collision avoidance**. To evaluate the conflict-avoidance effectiveness of the proposed path planning algorithm, we first analyze the temporal and spatial distribution of IAV node access frequencies under traffic demand 4. Figure 10 presents the visit count, from which both the temporal peak and the spatial congestion area can be identified. As shown in Figure 10a, the visit count peaks within the [360 s, 720 s] interval, reflecting the obvious traffic peak. Figure 10b further illustrates the node visit count across the yard layout in the peak period. The node number in the interval [1, 15] has the most intensive access, more than 300 times, representing the spatial congestion area. Figure 11 presents a node occupation timeline of 80 IAVs within the most congested time interval and node range. Each color represents a distinct IAV. The results show that no two IAVs simultaneously occupy the same node.

To further verify conflict-free routing across the entire network, Table 2 reports the average potential conflicts per IAV and the realized conflicts across all network cases. The average number of potential conflicts increases with traffic demand, from 0.33 under Demand 1 to 2.24 under Demand 4, with following conflicts occurring most frequently. Nevertheless, no realized conflicts are observed across the evaluated network cases, indicating that the generated routes satisfy the modeled conflict avoidance constraints under the tested conditions. Table 3 further presents the spatial distribution of potential conflicts across the six controlled intersections. The results indicate an uneven distribution, with potential conflicts becoming more concentrated at J1 and J2 as traffic demand increases. Their combined share rises from 39% under Demand 1 to 55% under Demand 4, suggesting that conflict exposure varies across intersection locations and demand levels.

**Computing efficiency**. Table 4 presents the IAV average delay and average computation time that the proposed path planning algorithm achieves under various traffic demands. As traffic intensity increases, the average delay rises significantly. This increase is primarily due to the growing number of potential conflicts at nodes and along paths, which elevates the complexity of feasible time window searches. The average computation time per IAV remains below 0.05 s across all scenarios, with a maximum of 0.04 s observed under the most congested case. This demonstrates the algorithm’s real-time applicability and scalability. Even under high traffic demand, the algorithm can provide timely routing updates, demonstrating its suitability for dynamic and time-constrained operations in ACTs.

### 5.3. Experiments on Platoon Strategy Based on Path Planning

In this section, the performance of the proposed platoon strategy based on path planning is compared with the FCFS strategy. The FCFS policy, which is widely used in most ports, assigns tasks to HTDs sequentially as they pass through intersections, based on their arrival order. Two scenarios are set up: a single intersection (as shown in Figure 3) and an IAV path network (as shown in Figure 9). The former scenario aims to assess the dynamic platoon strategy’s effectiveness, while the latter is designed to evaluate the efficiency of a comprehensive approach that integrates path planning and platoon strategies.

**Figure 12:**
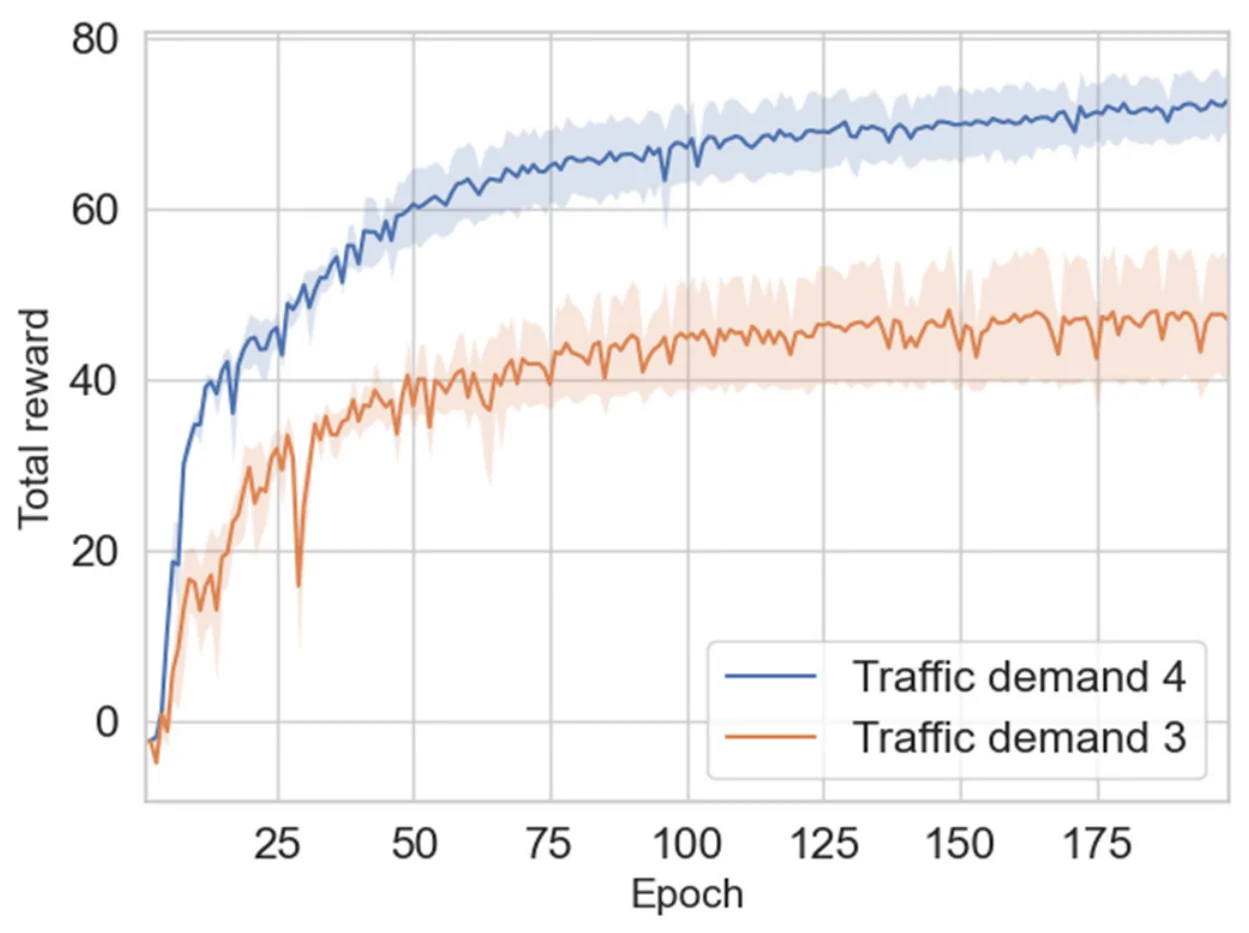
Convergence of the DQN algorithm in the single intersection scenario.

**Model convergence**. This study evaluates the learning performance of the proposed DQN algorithm under various traffic demands, specifically focusing on demands 3 and 4. Figure 12 shows the model’s consistent and stable learning results under both traffic demands. The convergence trend of the total reward is obvious under different conditions, which indicates that the model is highly adaptable to the dynamic traffic environment. It is critical to highlight that the convergence is slower under demand 4 than demand 3. This suggests that the agent requires more time to learn suitable strategies for adequate traffic flow management in more complex traffic scenarios.

**Figure 13:**
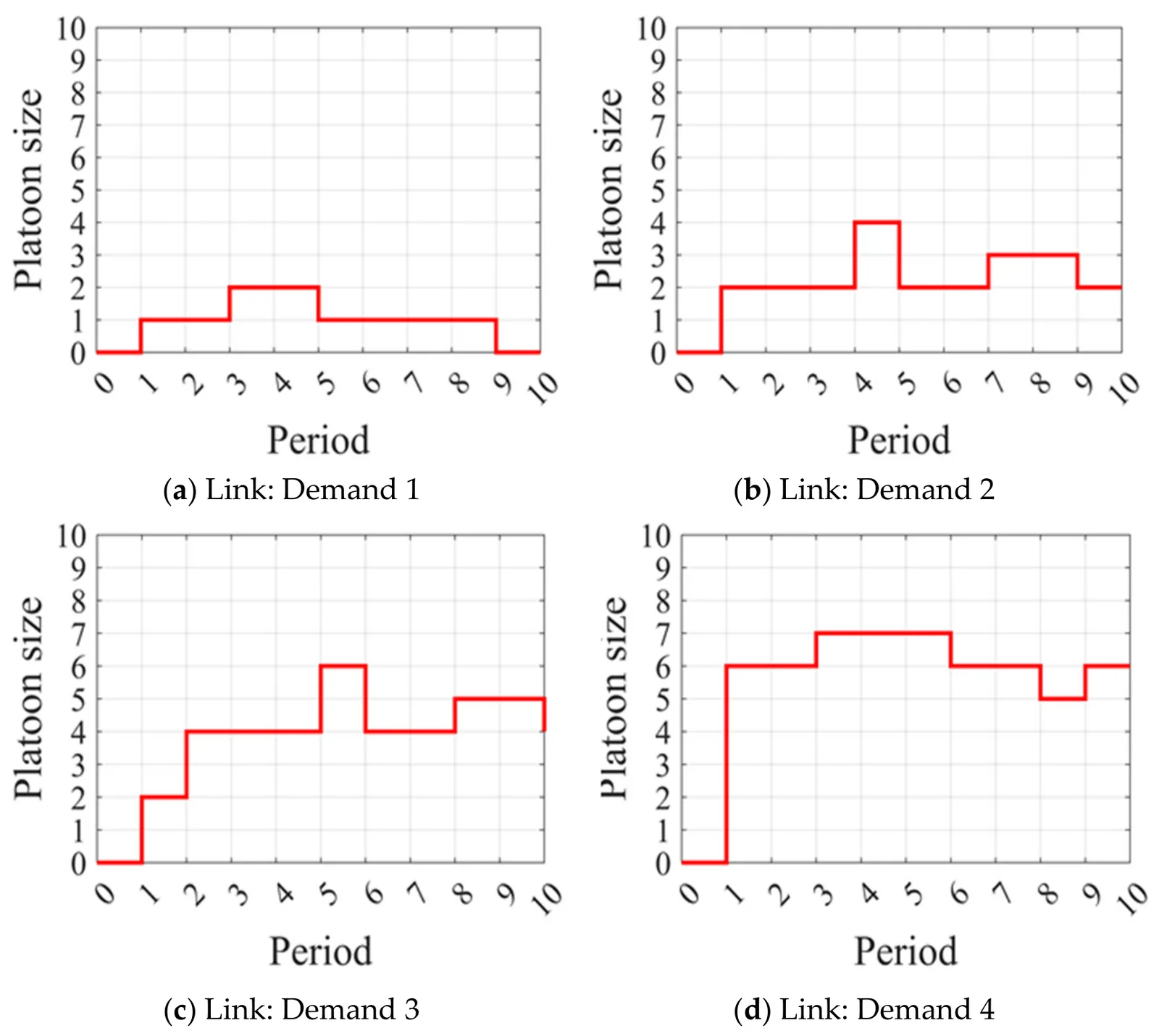
Platoon size in single link.

**Figure 14:**
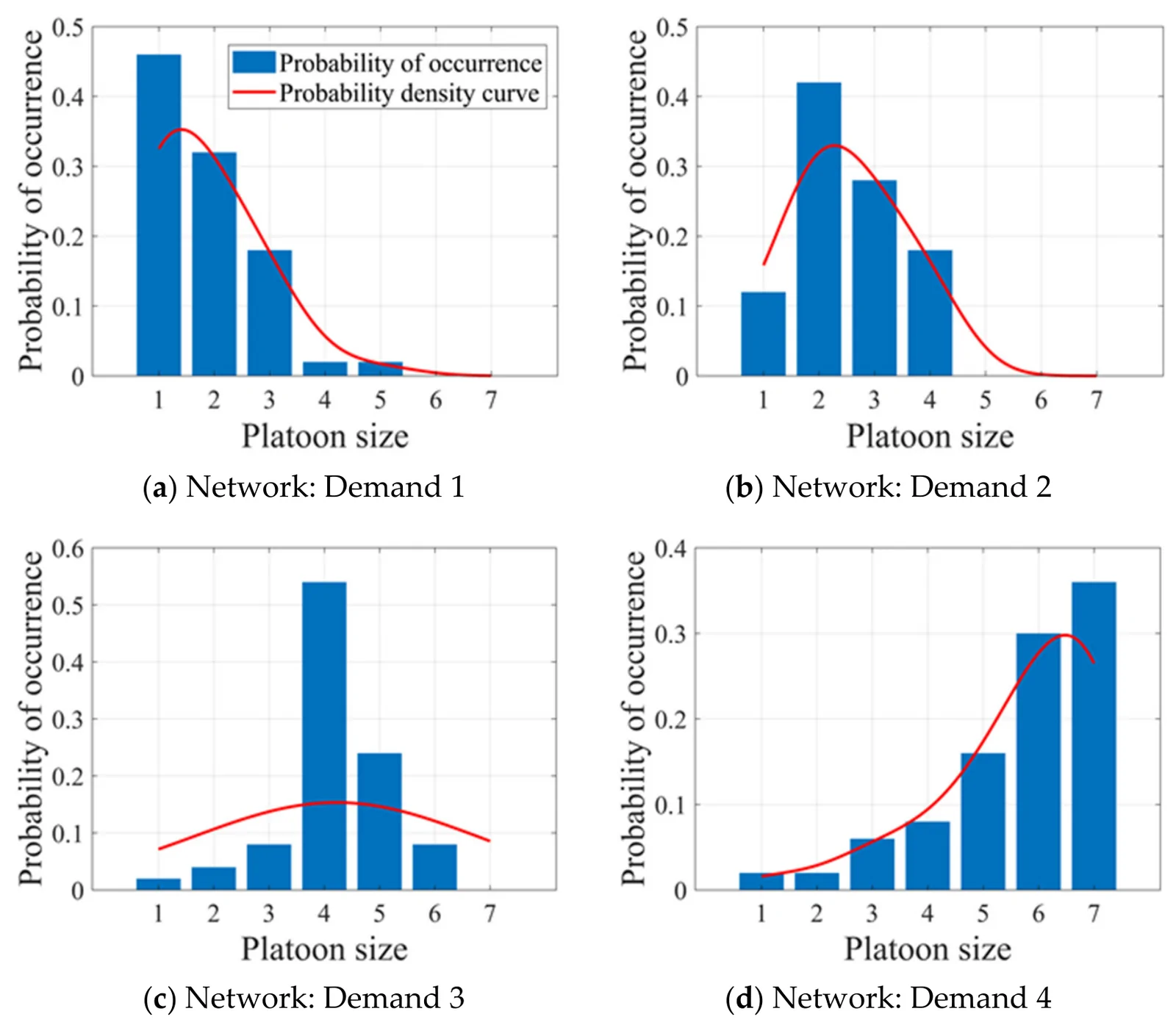
Probability distributions of platoon size.

**Decision adaptability**. Figure 13 illustrates the adjustment of platoon sizes on a single network link, capturing the dynamic optimization of platoon sizes within each period. This strategy can determine the optimal platoon size consistent with traffic flow fluctuations. Figure 14 further explores the probability distributions of platoon sizes across the entire network for varying traffic demands. The results show that in low-flow scenarios (e.g., demands 1 and 2), IAVs are more likely to form small sizes (1–2 IAVs) to keep traffic flowing and reduce unnecessary waiting times caused by formations. Conversely, under high traffic conditions (e.g., demands 3 and 4), larger sizes (5–6 IAVs) are formed more frequently to facilitate coordinated vehicle movement and reduce congestion at critical intersections. This dynamic adaptability reflects the model’s dual objectives: optimizing traffic flow and minimizing congestion-related delays at critical network nodes. By adjusting platoon sizes based on real-time traffic demands, the model adapts to varying traffic conditions, demonstrating its robust adaptability and efficiency in complex traffic environments. Its decision-making capability is evident at both the single-link and network-wide scales, where it adaptively adjusts platoon size to enhance overall traffic efficiency.

**Effectiveness comparison**. To further evaluate the effectiveness of the platoon strategy in handling various scale problems, this study conducts a comparison experiment of two intersection control strategies across two scenarios. The comparison, focusing on the average waiting time and delay presented in Table 5, reveals that the platoon strategy outperforms the FCFS strategy in most cases, particularly under medium and high traffic demand. It notably reduces the average delay and waiting time for IAVs at a single intersection and the entire ACT network. Specifically, the platoon strategy consistently outperforms FCFS in the single intersection scenario, particularly under medium and high traffic conditions. The maximum observed reductions in average delay and waiting time reach 34.45% and 31.31%, respectively. Similar advantages are evident in the network-level scenario, where the platoon strategy reduces average delay by up to 27.60% and waiting time by up to 38.09%. These improvements become increasingly evident as traffic demand intensifies, highlighting the strategy’s robustness in managing congested intersections and its adaptability to high-load operating environments.

However, improvements in the network scenario are not uniform across demand conditions or performance indicators. Specifically, in cases L1–L3 (Demand 1) and case L4 (Demand 2), the average delay is higher than that of FCFS by 72.61%, 34.33%, 38.37% and 2.72%, respectively, despite reductions in average waiting time. One possible explanation is that, when IAV arrivals are sparse, the time associated with platoon formation and coordination may outweigh the benefits of consecutive passage through intersections. In Case L8, the average waiting time increases by 33.33%, whereas the average delay is reduced by 13.43%. These results indicate that reductions in delay and waiting time are not necessarily achieved simultaneously. Additionally, to assess the computational efficiency of the integrated framework, computing time was evaluated for one network case at each demand level, namely L1, L4, L7 and L10. The average computing time was 22.10, 24.66, 35.08 and 43.12 ms, respectively. Although computing time increased with traffic demand, it remained low even at the highest demand level.

In conclusion, the RL-based platoon strategy exhibits strong adaptability across different traffic conditions. It offers a robust solution for intersection management in modern automated terminals, particularly in optimizing traffic flow and reducing IAV delays.

## 6. Conclusions and Discussions

This paper addresses the integrated problem of IAV path planning and intersection control in ACTs with a parallel layout. It proposes a dynamic platoon strategy that combines a space-time path planning algorithm with an RL algorithm to optimize routing and traffic management for IAVs.

Two simulation scenarios and four traffic demand levels were designed to evaluate the integrated approach. Extensive numerical experiments compare this approach with the conventional rule-based scheduling method for IAVs, demonstrating its effectiveness, particularly under higher traffic demand. The key conclusions are as follows: (1) A decentralized space-time path planning algorithm, Dijkstra-TW, was developed to consider IAV operational characteristics, terminal layout constraints, and dynamic node availability. This algorithm minimizes routing time while ensuring a safe and conflict-free route. (2) A deep RL-based platoon strategy was introduced to improve efficiency under high traffic demand. This adaptive strategy adjusts platoon sizes in real time, effectively alleviating congestion and improving crossing efficiency. (3) The integrated framework ensures IAV safety within the traffic network and significantly reduces average delays. It can reduce the average delay for IAVs by up to 34.45% in isolated intersection scenarios and 27.60% across the network, demonstrating significant potential for improving traffic management in ACTs.

Several issues warrant further investigation. The operational details of loading and unloading equipment are simplified, and the method should be examined in other terminal layouts. In addition, the present evaluation is based on simulation and does not establish the performance of a physical sensing or communication system. Future research should therefore (1) quantify how communication latency, packet loss and link failures affect delay and conflict-point utilization; (2) extend the framework to mixed traffic; and (3) use measured vehicle trajectories and other terminal layouts to assess how localization errors, observation delays and missing data affect occupancy timeline consistency and platoon control performance.

## Figures and Tables

**Table 1:** Traffic demand levels and sampled arrival rates in scenarios of the intersection and network.

Traffic Demand	Scenario					
	Intersection			Network		
	*ρ* Range (veh/h)	Case ID	Sampled *ρ* (veh/h)	*ρ* Range (veh/h)	Case ID	Sampled *ρ* (veh/h)
1	(0–200]	S1	112	(0–500]	L1	318
		S2	175		L2	405
		S3	104		L3	265
2	(200–400]	S4	236	(500–1000]	L4	582
		S5	293		L5	702
		S6	368		L6	916
3	(400–600]	S7	547	(1000–1500]	L7	1086
		S8	466		L8	1293
		S9	539		L9	1401
4	(600–800]	S10	702	(1500–2000]	L10	1937
		S11	683		L11	1638
		S12	741		L12	1712

**Table 2:** Potential and realized traffic conflicts across network scenarios.

Traffic Demand	Case ID	Average Potential Conflicts per IAV				Realized Conflicts
		Following	Crossing	Converging	Total	
1	L1	0.19	0.13	0.10	0.42	0
	L2	0.14	0.10	0.07	0.31	0
	L3	0.12	0.08	0.06	0.27	0
2	L4	0.29	0.21	0.17	0.68	0
	L5	0.34	0.27	0.20	0.81	0
	L6	0.46	0.38	0.27	1.12	0
3	L7	0.55	0.47	0.34	1.36	0
	L8	0.68	0.61	0.43	1.71	0
	L9	0.58	0.51	0.35	1.45	0
4	L10	0.93	0.85	0.60	2.38	0
	L11	0.86	0.78	0.56	2.21	0
	L12	0.83	0.76	0.55	2.14	0

Note: Potential conflicts are reported as the average number per IAV, whereas realized conflicts are reported as the total number of observed violations in each case. The verification covers all 102 nodes and the complete simulation horizon for each network case.

**Table 3:** Distribution of potential conflicts across the six controlled intersections under different traffic demand levels (%).

Intersection	Demand 1	Demand 2	Demand 3	Demand 4
J1	21	23	27	30
J2	18	22	23	25
J3	17	16	16	12
J4	15	14	11	12
J5	15	11	12	10
J6	14	14	11	11

**Table 4:** Average delays and average computing time of the path planning algorithm results.

Traffic Demand	Case ID	Average Delay (s)	Average Computing Time (s)
1	L1	9.96	0.01
	L2	5.91	0.01
	L3	4.74	0.01
2	L4	14.97	0.01
	L5	19.47	0.01
	L6	33.23	0.02
3	L7	41.43	0.02
	L8	58.23	0.02
	L9	44.28	0.02
4	L10	151.56	0.04
	L11	138.05	0.03
	L12	130.12	0.03

**Table 5:** Average delays and average waiting time comparison in two scenarios.

Scenario	Traffic Demand	Case ID	Average Delays		% Diff vs. FCFS	Average Waiting Time (s)		% Diff vs. FCFS
			FCFS	Platoon		FCFS	Platoon	
Intersection	1	S1	1.27	1.25	−1.57	0.38	0.35	−7.89
		S2	2.11	1.98	−6.16	0.37	0.34	−8.11
		S3	1.24	1.22	−1.61	0.40	0.32	−20.00
	2	S4	13.23	10.64	−19.58	1.41	1.27	−9.93
		S5	18.73	16.49	−11.96	1.44	1.31	−9.03
		S6	30.64	26.54	−13.38	1.47	1.25	−14.97
	3	S7	58.90	45.53	−22.70	3.54	2.88	−18.64
		S8	48.98	38.23	−21.95	3.35	2.68	−20.00
		S9	54.65	49.85	−8.78	3.69	3.00	−18.70
	4	S10	97.47	75.39	−22.65	7.87	5.43	−31.00
		S11	96.34	69.70	−27.65	7.01	5.44	−22.40
		S12	110.81	72.64	−34.45	7.89	5.42	−31.31
Network	1	L1	8.47	14.62	+72.61	0.45	0.41	−8.89
		L2	11.65	15.65	+34.33	0.48	0.44	−8.33
		L3	8.73	12.08	+38.37	0.45	0.42	−6.67
	2	L4	19.51	20.04	+2.72	1.99	1.74	−12.56
		L5	20.95	20.67	−1.34	2.09	1.78	−14.83
		L6	33.86	32.97	−2.63	1.99	1.83	−8.04
	3	L7	93.62	80.01	−14.54	5.81	4.00	−31.15
		L8	105.87	91.65	−13.43	4.56	6.08	+33.33
		L9	143.57	117.27	−18.31	6.48	2.24	−65.43
	4	L10	260.99	217.98	−16.48	19.90	12.32	−38.09
		L11	237.09	171.65	−27.60	11.87	9.93	−16.34
		L12	240.81	194.87	−19.08	12.98	8.91	−31.36

## Data Availability

Data will be made available on request.

## Appendix A. Model Notation

**Table A1:** Notation used in the space-time path planning model.

Notation	Description
*K*	set of IAVs
$K_{k}^{p}$	set of IAVs planned before IAV *k* under the FCFS planning order, $K_{k}^{p}\subset K$
*N*	set of all conflict nodes
*N^c^*	set of conflict nodes, excluding starting points and ending points
*N^m^*	set of crossing nodes and converging nodes
$N_{k^{\prime }}$	set of nodes on the planned path of IAV $k^{\prime }\in K_{k}^{p}$
*A*	set of directed arcs connecting nodes in *N*
*A^hand^*	set of handling lane arcs
*s_k_*, *e_k_*	start and end nodes of IAV, respectively
$T_{k}^{\mathrm{gen}}$	task release time of IAV *k*
*d_ij_*	length of arc (*i*,*j*)
*v*_empty_	maximum speed of an empty IAV
*v*_load_	maximum speed of a loaded IAV
*s_k_*_,*ij*_	load state of IAV *k* on arc (*i*,*j*), taking the value empty or load
*τ_i_*	service/occupation time at node *i*, *τ_i_* = *τ*^1^ if *i* ∈ *N^c^* and *τ_i_* = *τ*^2^ if *i* ∈ *N*∖*N^c^*
$b_{i}^{1}$	safety buffer (minimum time headway between two IAVs) under IAV following conflict at node *i*
$b_{i}^{2}$	safety buffer (minimum time headway between two IAVs) under IAV crossing or converging conflict at node *i*
*M*	a sufficiently big constant
*a_k_*_,*i*_	arrival time of IAV *k* at node *i*
*β_k_*_,*ij*_	entry time of IAV *k* into arc (*i*,*j*)
*δ* *_k_*_,*ij*_	equals 1 if IAV *k* enters arc (*i*,*j*), and 0 otherwise.
$z_{k,k^{\prime },i}$	equals 1 if IAV *k* passes node *i* after IAV *k*′, 0 otherwise
$w_{k,k^{\prime },(i,j)}$	equals 1 if IAV *k* enters arc (*i*,*j*) after IAV *k*′ has left it, 0 otherwise

## Appendix B. Experimental Parameter Settings

**Table A2:** Terminal layout, vehicle operating, and DQN parameters used in the numerical experiments.

Category	Parameter	Value
Terminal layout	Length of horizontal transportation area	1200 m
	Width of horizontal transportation area	1000 m
	The number of berths	4
	The number of stacks	12
Vehicle operation	Loaded speed of IAV	7 m/s
	Empty speed of IAV	10 m/s
DQN	Learning rate	0.0001
	Discount factor *γ*	0.99
	Hidden-layer sizes	[256, 512, 256, 128]
	Training Epoch	1000
	Training time step	60 s
	Batch size	32
	Replay memory capacity	20,000
	Target network update interval	50
	Exploration rate	0.1 → 0.01
	Warm up period	3
	Optimizer	Adam
	Loss function	Mean square error (MSE)
